# SDF-1α Promotes Chondrocyte Autophagy through CXCR4/mTOR Signaling Axis

**DOI:** 10.3390/ijms24021710

**Published:** 2023-01-15

**Authors:** Jiazhou Li, Hao Chen, Lang Cai, Daimo Guo, Demao Zhang, Xuedong Zhou, Jing Xie

**Affiliations:** 1 State Key Laboratory of Oral Diseases, West China Hospital of Stomatology, Sichuan University, Chengdu 610041, China; 2 National Clinical Research Center for Oral Diseases, West China Hospital of Stomatology, Sichuan University, Chengdu 610041, China; 3 Department of Cariology and Endodontics, West China Hospital of Stomatology, Sichuan University, Chengdu 610041, China

**Keywords:** SDF-1α, autophagy, chondrocyte, CXCR4, mTOR

## Abstract

SDF-1α, the most common isoform of stromal cell-derived factor 1, has shown vital effects in regulating chondrocyte proliferation, maturation, and chondrogenesis. Autophagy is a highly conserved biological process to help chondrocytes survive in harsh environments. However, the effect of SDF-1α on chondrocyte autophagy is still unknown. This study aims to investigate the effect of SDF-1α on chondrocyte autophagy and the underlying biomechanism. Transmission electron microscope assays and mRFP-GFP-LC3 adenovirus double label transfection assays were performed to detect the autophagic flux of chondrocytes. Western blots and immunofluorescence staining assays were used to detect the expression of autophagy-related proteins in chondrocytes. RNA sequencing and qPCR were conducted to assess changes in autophagy-related mRNA expression. SDF-1α upregulated the number of autophagosomes and autolysosomes in chondrocytes. It also increased the expression of autophagy-related proteins including ULK-1, Beclin-1 and LC3B, and decreased the expression of p62, an autophagy substrate protein. SDF-1α-mediated autophagy of chondrocytes required the participation of receptor CXCR4. Moreover, SDF-1α-enhanced autophagy of chondrocytes was through the inhibition of phosphorylation of mTOR signaling on the upstream of autophagy. Knockdown by siRNA and inhibition by signaling inhibitor further confirmed the importance of the CXCR4/mTOR signaling axis in SDF-1α-induced autophagy of chondrocytes. For the first time, this study elucidated that SDF-1α promotes chondrocyte autophagy through the CXCR4/mTOR signaling axis.

## 1. Introduction

Stromal cell-derived factor 1 (SDF-1), also known as CXC motif chemokine ligand 12 (CXCL12), is a member of the chemoattractant cytokine superfamily [1,2]. Recently, it has been defined as a dual-use homeostatic/inflammatory cytokine [3]. For instance, SDF-1 can help to maintain tissue homeostasis while also supporting inflammatory cell infiltration [3,4]. SDF-1α is the most common and most widely recognized isoform of SDF-1 and has been shown to play an essential role in cellular behaviors, including cell proliferation [5], distribution [6], differentiation [7] and apoptosis [8]. A previous report also indicated that it was potentially related to autophagy [9], but the underlying biological effect of SDF-1α on autophagy is still largely unknown.

Autophagy is a highly conserved cytoprotective mechanism that maintains cell homeostasis by degrading redundant cytoplasmic materials and restructuring new components [10]. Currently, autophagy is classified into three main categories: macroautophagy, microautophagy and chaperone-mediated autophagy [11]. Under normal circumstances, autophagy proceeds at basal levels to keep cells functioning well [12]. Meanwhile, autophagy can be induced under stress, such as oxygen deficit or nutritional deprivation, to help cell survival [13]. In mammals, the autophagic program is initiated at multiple sites in cells. It undergoes induction and nucleation, and expands to form a phagophore [14]. Next, the membrane elongates and fully engulfs the cargoes, including unwanted cytosolic components, damaged or superfluous organelles, lipid droplets, etc., and the double-membrane autophagosomes are formed [15]. In order to degrade the selected cargoes, the outer membrane of autophagosomes first fuses with lysosomes, referred to as autolysosomes [16], to wrap up the cargoes. The hydrolytic enzymes in lysosomes then degrade the inner membrane and all these cargoes, consequentially allowing the degradation products to be released into the cytoplasm for the resynthesis of biomacromolecules or into other metabolic pathways [17]. The ability of autophagy in recycling and reusing can help cells adapt to stress conditions and temporarily survive in harsh environments.

Chondrocytes, the sole kind of cell in cartilage tissue, are essential to preserving the homeostasis of cartilage tissue [18]. However, the homeostasis of cartilage tissue is readily disturbed because of the unique organizational features of cartilage tissue—no blood vessels, nerves or lymphatics [19]. Osteoarthritis (OA), one of the most typical cartilage diseases, is carried on by an imbalance in cartilage homeostasis and simultaneously alters chondrocyte behaviors including proliferation, migration, differentiation and apoptosis [4,20]. Recent research has begun to reveal the sharp rise of SDF-1 concentration around OA cartilage and the potential implications of SDF-1 on chondrocyte cell behaviors involving chondrocyte differentiation, proliferation and catabolism [21]. However, the effect of SDF-1 on chondrocyte autophagy and its underlying biomechanism are still unknown. Therefore, elucidating the role of SDF-1 on chondrocyte autophagy might provide new perspectives on the initiation of cartilage disease and new potentials for the treatment of cartilage diseases.

In the present study, we aimed to use recombinant SDF-1α to explore the effect on chondrocyte autophagy. Based on RNA sequencing, the changes in autophagy levels were comprehensively analyzed by western blot assays, immunofluorescence staining assays, mRFP-GFP-LC3 adenovirus double label assays and transmission electron microscope (TEM) assays. These results help us to understand the importance of SDF-1α on autophagy of chondrocytes.

## 2. Results

### 2.1. SDF-1α Promotes Autophagy Phenotype in Chondrocytes

To determine the potential role of SDF-1α on the chondrocyte phenotype, we firstly screened all gene subtypes of protein markers of cartilage phenotype from RNA sequencing (Figure S1 and Table S4) and detected the expression of collagen type II and aggrecan by western blots (Figure S2), and confirmed that SDF-1α did not significantly change the chondrocyte phenotype after treatment for 48 h.

**Figure 1:**
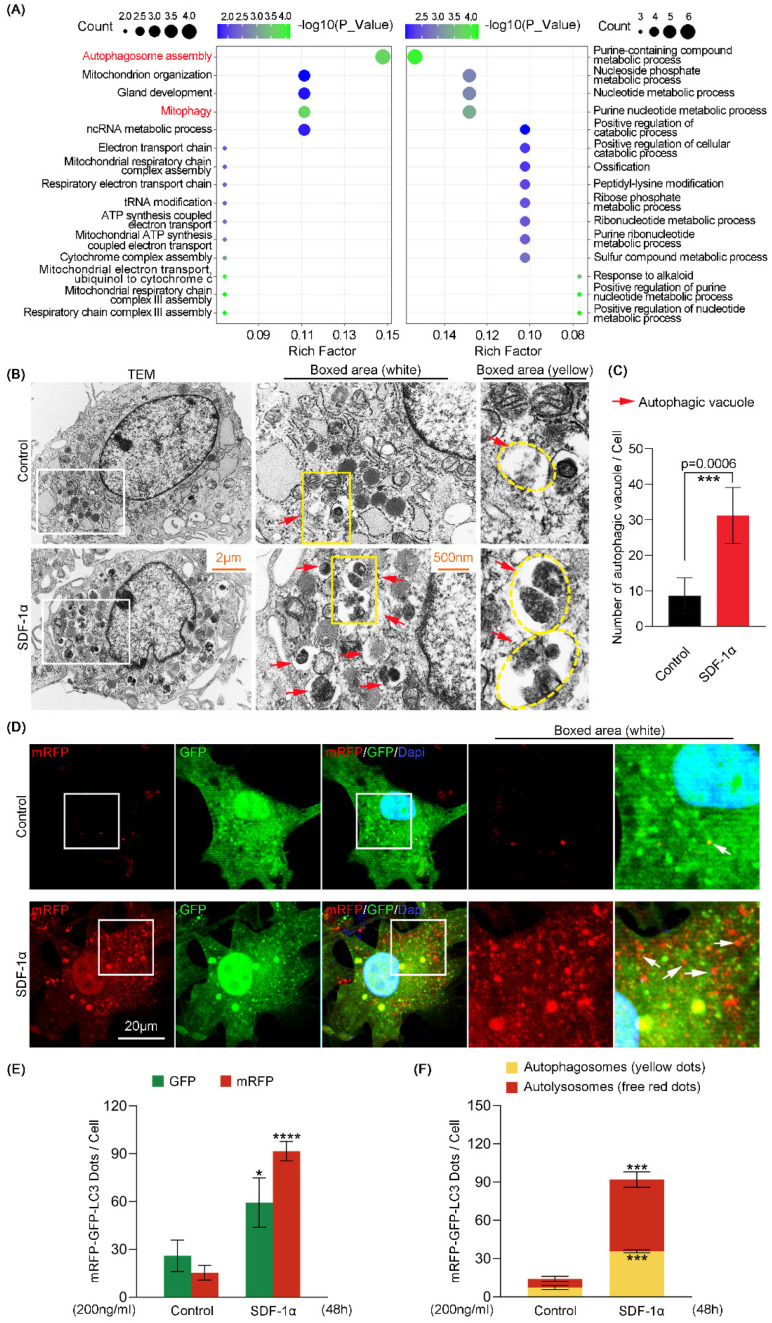
SDF-1α promotes autophagy phenotype in chondrocytes. (**A**) Gene ontology (GO) enrichment based on RNA sequencing demonstrating autophagy-related biological processes (BP) in chondrocytes induced by SDF-1α (200 ng/mL) for 48 h. (**B**) Representative TEM images showing that the number of autophagic vacuoles was increased in chondrocytes after treatment with 200 ng/mL SDF-1α for 24 h. Red arrows indicate autophagic vacuole. (**C**) Quantitative analysis of the number of autophagic vacuoles shown in (**B**). We counted five cells from three independent experiments in each group to demonstrate the number of autophagic vacuoles. (**D**) mRFP-GFP-LC3 adenovirus double label assay indicating the increase of autophagy flux in chondrocytes after treatment with 200 ng/mL SDF-1α for 48 h. Free red dots represent autolysosomes. Yellow dots represent autophagosomes. White arrows point to the autolysosome. We counted six cells from three independent experiments in each group to detect the changes in the levels of autophagy. (**E**) Quantitative analysis of the number of mRFP and GFP dots in chondrocytes shown in (**D**). (**F**) Quantitative analysis of the number of autophagosomes and autolysosomes in chondrocytes shown in (**D**). The data shown in (**A**,**B**,**D**) are based on three independent experiments (*n* = 3). Data in (**C**,**E**,**F**) are presented as the mean ± SD, and the significance is based on two-tailed Student’s *t*-tests. * *p* < 0.05; *** *p* < 0.001; **** *p* < 0.0001.

To determine the evidence for the potential role of SDF-1α on the autophagy pathway, we performed Gene Ontology (GO) enrichment analysis based on RNA sequencing. The results about autophagy-related biological processes (BP) in GO enrichment directly indicated the upregulation of two processes, autophagosome assembly and mitophagy (Figure 1A and Table S1). To directly observe the autophagy phenotype in chondrocytes induced by SDF-1α, we performed TEM tests and found that the number of autophagic vacuoles (red arrow) was significantly increased in chondrocytes with the treatment of SDF-1α at 200 ng/mL for 24 h (Figure 1B). Quantification of autophagic vacuoles further confirmed this result (Figure 1C). Furthermore, we used mRFP-GFP-LC3 adenovirus double label assays to detect the changes in the levels of autophagy in chondrocytes induced by SDF-1α (200 ng/mL) for 48 h (Figure 1D). The yellow dots resulting from the overlap of mRFP (red) and GFP (green), suggest the formation of autophagosomes due to co-expressions of mRFP, GFP and microtubule-associated protein 1 light chain 3 (LC3), a classical autophagy marker in individual chondrocytes [22]. Later, autophagosomes (yellow) fuse with lysosomes to form autolysosomes, in which the local acidic environment generated makes GFP (green) quench and only mRFP (red) remain. This process of forming autolysosomes enables the original substances in autophagy to be degraded and reused, so as to repair and rebalance the intracellular homeostasis, realizing the function of the autophagy process [23]. After treatment with SDF-1α, we first found that more autophagosomes (yellow dots) were formed in chondrocytes than those in the non-treated normal group. Meanwhile, we observed that a large number of autolysosomes (red dots) were formed in chondrocytes relative to those in the non-treated normal group, indicating the importance of SDF-1α in positive functional autophagy process (Figure 1D). The quantitative analysis of autophagic flux further confirmed these results (Figure 1E,F). Taken together, we initially proved that SDF-1α could promote autophagy phenotype in chondrocytes.

### 2.2. SDF-1α Induces the Expression of Autophagy-Related Proteins in Chondrocytes

**Figure 2:**
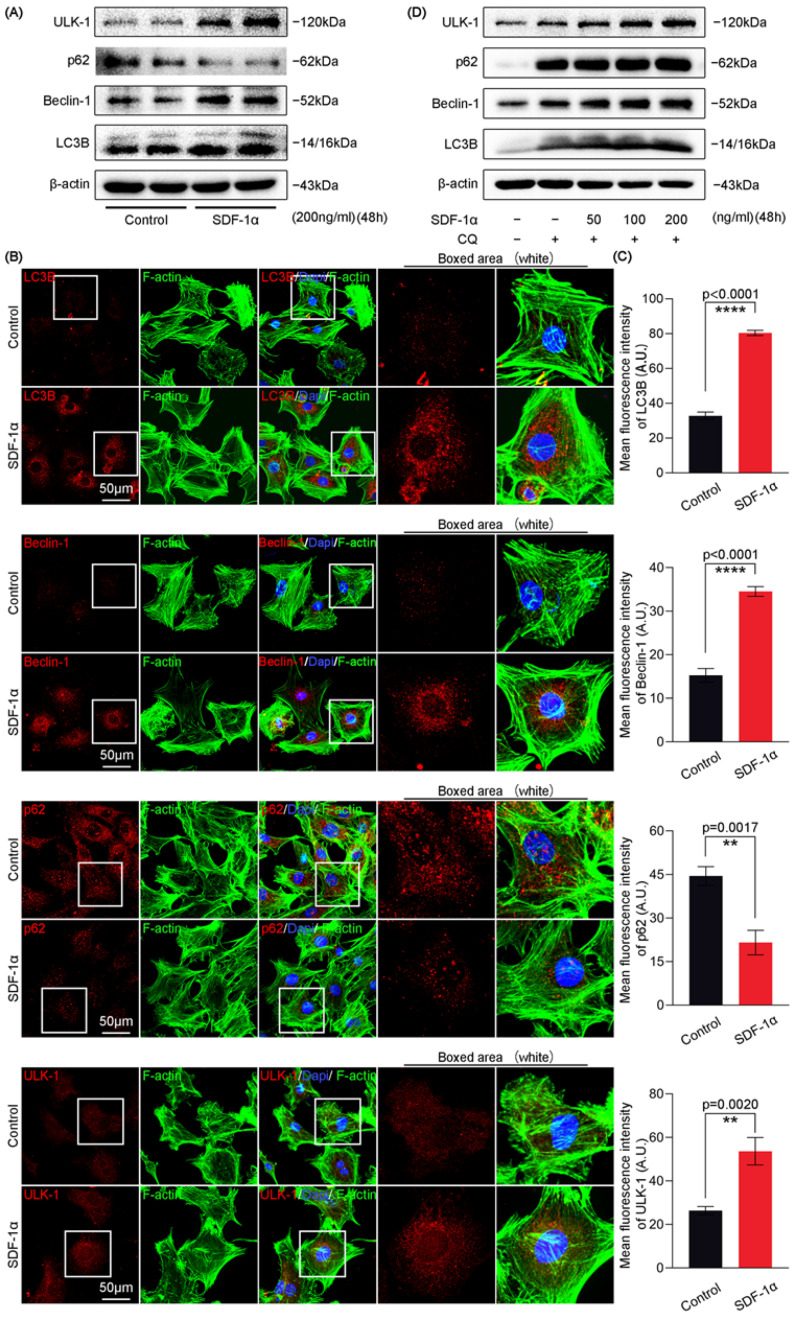
SDF-1α increases the expression of autophagy-related proteins in chondrocytes. (**A**) Representative western blots showing that the expression of ULK-1, Beclin-1 and LC3B increased, and that the expression of p62 decreased after treatment with SDF-1α (200 ng/mL) for 48 h. (**B**) Representative immunofluorescent images indicating the expression and distribution of ULK-1, p62, Beclin-1 and LC3B in chondrocytes induced by SDF-1α (200 ng/mL) for 48 h. (**C**) Quantification of the mean fluorescence intensity of autophagy-related proteins in chondrocytes shown in (**B**). We counted 15 cells per group from three independent experiments to show the changes in the expression and distribution of autophagy-related proteins. (**D**) Representative western blots showing the expression of ULK-1, p62, Beclin-1 and LC3B in chondrocytes induced by SDF-1α (0, 50, 100 and 200 ng/mL) in the presence of CQ (20 μM) for 48 h. The data shown in (**A**,**B**,**D**) are based on three independent experiments (*n* = 3). Data in (**C**) are presented as the mean ± SD, and the significance is based on two-tailed Student’s *t*-tests. ** *p* < 0.01; **** *p* < 0.0001.

To verify the effect of SDF-1α on chondrocyte autophagy at the protein level, we performed western blots to detect the vital proteins including ULK-1 [24], p62 [25], Beclin-1 [26] and LC3B [27] in the autophagy pathway. We found that the expression of ULK-1, Beclin-1 and LC3B was upregulated and that the expression of p62, an autophagy substrate protein, was downregulated in chondrocytes with the treatment of SDF-1α at 200 ng/mL for 48 h (Figure 2A). Quantitative analysis confirmed the expression changes of these proteins (Figure S3). To directly observe the expression and distribution of autophagy-related proteins in chondrocytes induced by SDF-1α, we performed immunofluorescence staining. By using CLSM, we found that the expression of ULK-1, Beclin-1 and LC3B was increased, and the expression of p62 was decreased in chondrocytes induced by SDF-1α (Figure 2B). The mean fluorescence intensity quantification further confirmed these results (Figure 2C). To further confirm the actual level of autophagy induced by SDF-1, we utilized chloroquine (CQ), an autophagy inhibitor. CQ acts at the degradation stage and can inhibit not only lysosome function but also the fusion between lysosomes and autophagosomes, leading to an accumulation of autophagy-related proteins [28]. We performed western blots to detect the expression of autophagy-related proteins in chondrocytes induced by SDF-1α in the presence of CQ (20 μM). The results show that CQ could enhance the expressions of autophagy markers, i.e., ULK-1, Beclin-1 and LC3B, and autophagic substrate marker p62 in chondrocytes. With the treatment of SDF-1α, the expression of these autophagy markers and the autophagic substrate marker were further enhanced in chondrocytes in the presence of CQ (Figure 2D). Quantitative analysis confirmed the expression changes of these proteins (Figure S4). Collectively, these results regarding autophagy-related proteins indicate the enhanced autophagy at the protein level in chondrocytes induced by SDF-1α.

### 2.3. SDF-1α Promotes Autophagy of Chondrocytes through CXCR4

**Figure 3:**
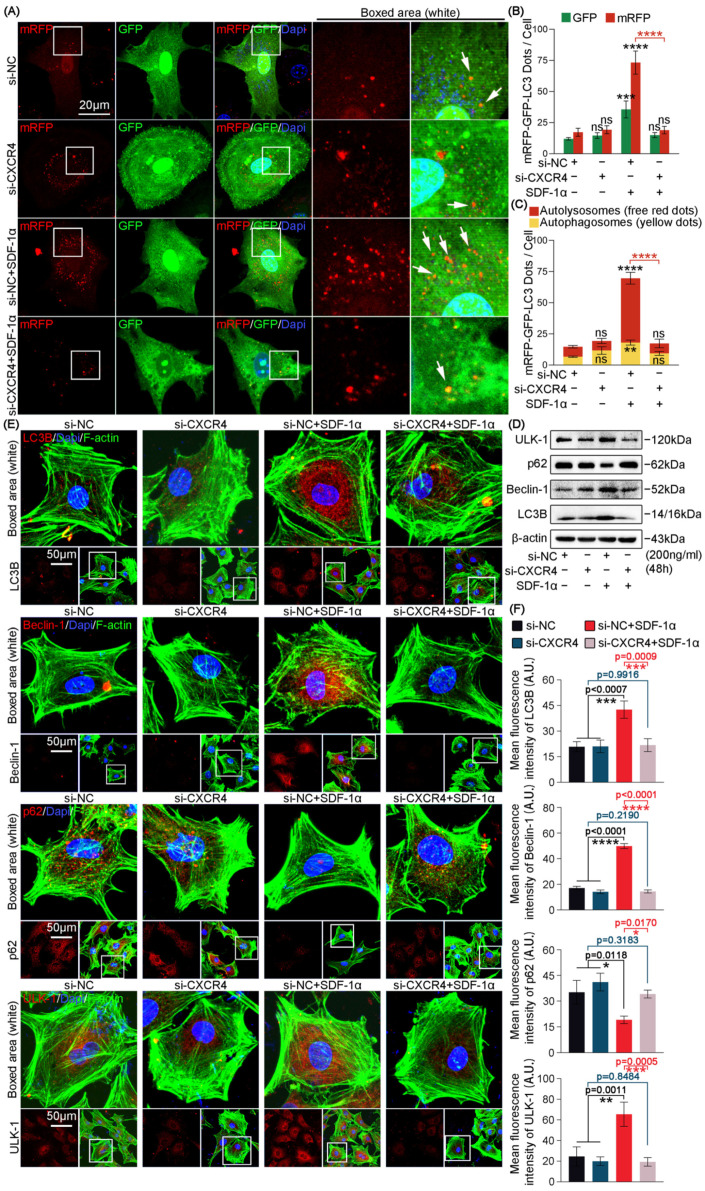
SDF-1α regulates autophagy of chondrocytes through CXCR4. (**A**) mRFP-GFP-LC3 adenovirus double label assay indicating that CXCR4 silencing inhibited the increase of autophagy flux in chondrocytes induced by SDF-1α (200 ng/mL) for 48 h. Free red dots represent autolysosomes. Yellow dots represent autophagosomes. White arrows point to the autolysosome. We counted three cells from three independent experiments in each group to detect the changes in the levels of autophagy. (**B**) Quantitative analysis of the number of mRFP and GFP dots in chondrocytes shown in (**A**). (**C**) Quantitative analysis of the number of autophagosomes and autolysosomes in chondrocytes shown in (**A**). (**D**) Representative western blots showing that si-CXCR4 inhibited the expression changes of autophagy-related proteins (ULK-1, p62, Beclin-1 and LC3B) induced by SDF-1α (200 ng/mL) for 48 h. (**E**) Representative immunofluorescent images demonstrating that the expression changes of autophagy-related proteins (ULK-1, p62, Beclin-1, LC3B) induced by SDF-1α (200 ng/mL) for 48 h were inhibited in chondrocytes after CXCR4 silencing. Cytoskeleton (F-actin), green; Nucleus (Dapi), blue (**F**) Quantitative analysis of the mean fluorescence intensity of ULK-1, p62, Beclin-1 and LC3B in chondrocytes shown in (**E**). We counted 15 cells per group from three independent experiments to show the changes in the expression and distribution of autophagy-related proteins. The data shown in (**A**,**D**,**E**) are based on three independent experiments (*n* = 3). Data in (**B**,**C**,**F**) are presented as the mean ± SD, and the significance is based on two-tailed Student’s *t*-tests. ns: no statistical significance; * *p* < 0.05; ** *p* < 0.01; *** *p* < 0.001; **** *p* < 0.0001.

There are two receptors for SDF-1α, CXCR4 and CXCR7, expressed on the cell membrane of chondrocytes [29]. In comparison, the expression of CXCR7 was much lower than CXCR4 in our results of RNA sequencing (Table S2). Moreover, CXCR4 is generally considered to be the driver of the biological effects of SDF-1α, compared to CXCR7 [4]. To verify the potential role of SDF-1α/CXCR4 axis on the autophagy pathway, we designed the following experiments. We designed three different siRNA sequences targeting CXCR4 to knock down its expressions and found that all these three siRNA sequences could successfully decrease the expression of CXCR4 (Figure S5). In the remaining experiments, we selected the siRNA-3 (50 nM), which showed the highest knockdown efficiency of CXCR4. To detect the role of CXCR4 in autophagy phenotype in chondrocytes induced by SDF-1α, we performed mRFP-GFP-LC3 adenovirus double label assays (Figure 3A). The results show that CXCR4 knockdown greatly impaired the increased number of autophagosomes (yellow) and autolysosomes (red) in chondrocytes induced by SDF-1α (Figure 3A). Quantitative analysis confirmed the impairment of autophagosome and autolysosome in chondrocytes induced by SDF-1α after si-CXCR4 transfection (Figure 3B,C). This mRFP-GFP-LC3 adenovirus double label assay indicated that SDF-1α-induced autophagic flux required the participation of CXCR4. To verify the influence of CXCR4 in the expression of autophagy-related proteins, we performed western blots and immunofluorescence staining (Figure 3D,E). From western blots, we found that the protein expression of autophagy markers, i.e., ULK-1, Beclin-1 and LC3B was decreased, and the expression of autophagic substrate marker p62 was increased in SDF-1α-induced chondrocytes after si-CXCR4 transfection, relative to those of the SDF-1α-treated group (Figure 3D). Quantitative analysis confirmed the expression changes of these autophagy-related proteins (Figure S6). From immunofluorescence staining, we confirmed that si-CXCR4 decreased the cytoplasmic expression of ULK-1, Beclin-1 and LC3B and increased the expression of p62 in chondrocytes induced by SDF-1α (Figure 3E). The quantification of mean fluorescence intensity confirmed these results (Figure 3F). Overall, we revealed that SDF-1α promotes autophagy via CXCR4.

### 2.4. SDF-1α Mediates Autophagy of Chondrocytes through Inhibiting the Phosphorylation of mTOR

**Figure 4:**
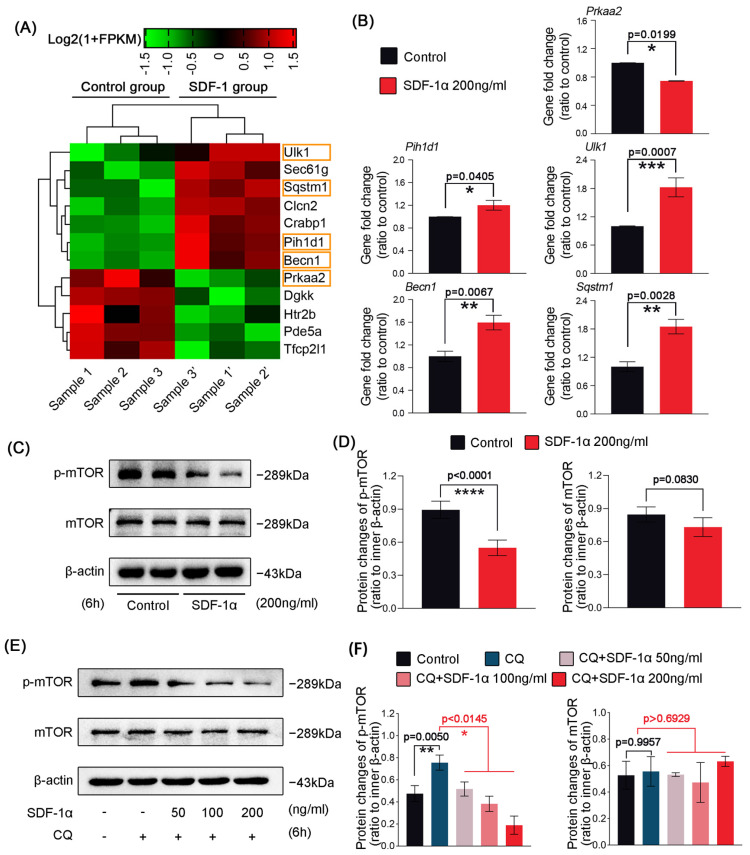
SDF-1α inhibits the mTOR/p-mTOR signaling in chondrocytes (**A**) Pheatmap based on RNA sequencing indicating the mRNA changes of autophagy pathway in chondrocytes in response to SDF-1α (200 ng/mL) for 48 h. The datasets are presented as FPKM (Fragments Per Kilobase of transcript per Million fragments mapped) by online R-package from RNA sequencing data. Three independent pairs of samples, i.e., Sample 1 and 1′, Sample 2 and 2′, and Sample 3 and 3′, were from the same mother cells, respectively. (**B**) qPCR confirming the mRNA changes of Pih1d1, Ulk1, Becn1, Sqstm1 and Prkaa2 in chondrocytes in response to SDF-1α (200 ng/mL). GAPDH gene was used as the inner control. (**C**) Representative western blots showing the expression of mTOR and p-mTOR in chondrocyte treated with SDF-1α (200 ng/mL) for 6 h. (**D**) Quantitative analysis of the expression changes of mTOR and p-mTOR shown in (**C**). (**E**) Representative western blots showing the expression of mTOR and p-mTOR in chondrocyte treated with 200 ng/mL SDF-1α for 6 h in the presence or absence of CQ (20 μM). SDF-1α inhibited phosphorylation of mTOR in chondrocytes in a dose-dependent manner. (**F**) Quantitative analysis of the expression changes of mTOR and p-mTOR shown in (**E**). The data shown in (**A**–**C**,**E**) are based on three independent experiments (*n* = 3). Data in (**B**,**D**,**F**) are presented as the mean ± SD. The significance is based on one-way analysis of variance. * *p* < 0.05; ** *p* < 0.01; *** *p* < 0.001; **** *p* < 0.0001.

To check the specific change of gene candidates in autophagy-related biological processes of GO enrichment in Figure 1A, we screened RNA sequencing data and clustered a pheatmap by online R-package (Figure 4A and Table S3). Among these candidates, we found that genes such as autophagy activating kinase 1 (Ulk1), beclin 1 (Becn1) and sequestosome 1 (Sqstm1), which are typically responsible for autophagy [30,31,32], were all upregulated. At the same time, we also found that genes such as PIH1 domain containing 1 (Pih1d1) and 5′-AMP-activated protein kinase catalytic subunit alpha-2 (Prkaa2), which are directly related to mTOR signaling [33,34], were changed. The qPCR results further confirmed the change in these major representative genes (Figure 4B). As the above data show, mTOR signaling, which promotes biomolecule synthesis and simultaneously inhibits autophagy [35], might be involved in chondrocyte autophagy induced by SDF-1α. Thus, we performed western blots to check the protein change of mTOR signaling (Figure 4C). The results show that the total expression of mTOR was not changed, but the phosphorylation of mTOR was significantly decreased in chondrocytes with the treatment of SDF-1α at 200 ng/mL for 6 h (Figure 4C,D). These results suggest that the effect of SDF-1α on the mTOR signaling pathway might be a post-translational modification. By using autophagy inhibitor CQ (20 μM), we first found the upregulation of p-mTOR signaling in normal chondrocytes, however, its expression was sharply decreased in chondrocytes in the presence of SDF-1α at 200 ng/mL for 6 h (Figure 4E,F). At the same time, the protein expression of p-mTOR decreased in a dose-dependent manner induced by SDF-1α in the presence of CQ (20 μM), and the expression of total mTOR showed no significant changes (Figure 4E). Together, these data indicate that SDF-1α mediates autophagy via inhibiting phosphorylation of mTOR on the upstream signaling of autophagy.

### 2.5. SDF-1α Regulates Autophagy of Chondrocytes through the CXCR4/mTOR Signaling Axis

**Figure 5:**
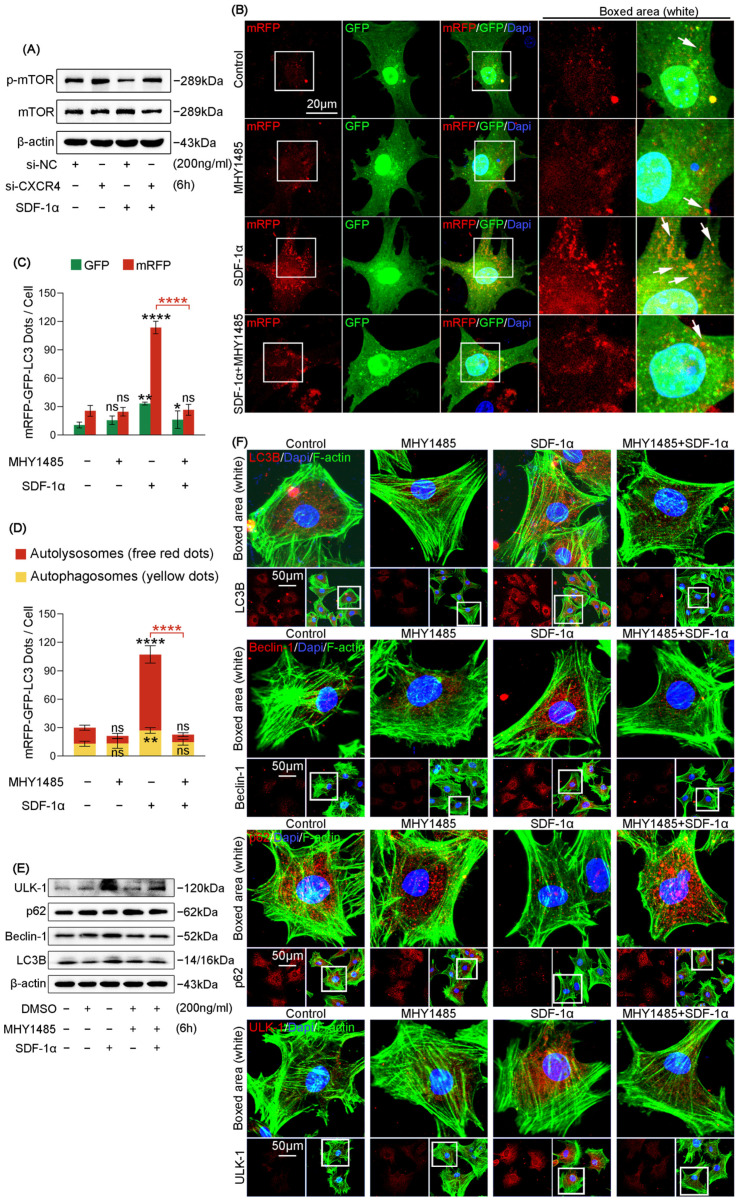
SDF-1α enhances autophagy through inhibition of mTOR/p-mTOR signaling in chondrocytes (**A**) Representative western blots showing the expression of mTOR and p-mTOR in chondrocyte treated with SDF-1α (200 ng/mL) for 6 h in the presence or absence of CXCR4 siRNA (50 nM). (**B**) mRFP-GFP-LC3 adenovirus double label assay indicating that MHY1485 (100 nM) impaired the increase of autophagy flux in chondrocytes induced by SDF-1α (200 ng/mL) for 48 h. Free red dots represent autolysosomes. Yellow dots represent autophagosomes. White arrows point to the autolysosome. We counted three cells from three independent experiments in each group to detect the changes in the levels of autophagy. (**C**) Quantitative analysis of the number of mRFP and GFP dots in chondrocytes shown in (**B**). (**D**) Quantitative analysis of the number of autophagosomes and autolysosomes in chondrocytes shown in (**B**). (**E**) Representative western blots showing that MHY1485 (100 nM) inhibited the expression changes of autophagy-related proteins (ULK-1, p62, Beclin-1 and LC3B) in chondrocytes induced by SDF-1α (200 ng/mL). (**F**) Representative immunofluorescent images demonstrating that the expression changes of autophagy-related proteins (ULK-1, p62, Beclin-1 and LC3B) in chondrocytes induced by SDF-1α (200 ng/mL) were inhibited after pretreatment with MHY1485 (100 nM). Cytoskeleton (F-actin), green; nucleus (Dapi), blue. We counted 15 cells per group from three independent experiments to show the changes in the expression and distribution of autophagy-related proteins. The data shown in (**A**,**B**,**E**,**F**) are based on three independent experiments (*n* = 3). Data in (**C**,**D**) are presented as the mean ± SD, and the significance is based on one-way analysis of variance. ns: no statistical significance; * *p* < 0.05; ** *p* < 0.01; **** *p* < 0.0001.

To check the interdependence between CXCR4 and mTOR signaling in SDF-1α-mediated autophagy in chondrocytes, we first performed si-CXCR4 transfection to detect the expression of mTOR signaling (Figure 5A). The results showed that si-CXCR4 increased the SDF-1α-induced downregulation of p-mTOR signaling. Quantitative analysis also confirmed the expression changes (Figure S7). These results indicate the correlation between CXCR4 and mTOR signaling in chondrocytes induced by SDF-1α. To determine the role of p-mTOR signaling in SDF-1α-mediated autophagy, we used MHY1485, an activator of phosphorylation activation of mTOR [36]. From mRFP-GFP-LC3 adenovirus double label assays, we found that MHY1485 could greatly impair the increased number of autophagosomes (yellow) and autolysosomes (red) in chondrocytes induced by SDF-1α (Figure 5B). Quantitative analysis confirmed the impairment of autophagosome and autolysosome in chondrocytes induced by SDF-1α after treatment with MHY1485 (Figure 5C,D). This mRFP-GFP-LC3 adenovirus double label assay indicated the importance of mTOR signaling in SDF-1α-induced autophagic flux. From western blots, we found MHY1485 decreased the expression of autophagy-related proteins, i.e., ULK-1, Beclin-1 and LC3B in chondrocytes enhanced by SDF-1α, and restored the expression of autophagic substrate marker p62 in chondrocytes reduced by SDF-1α (Figure 5E and Figure S8). From immunofluorescence staining, we explored the expression and distribution changes of these proteins (Figure 5G). MHY1485 decreased the cytoplasmic expression of ULK-1, Beclin-1 and LC3B in chondrocytes enhanced by SDF-1α and restored the expression of autophagic substrate marker p62 in chondrocytes reduced by SDF-1α. The quantification of mean fluorescence intensity further confirmed these results (Figure S9). Taken together, the evidence above indicates the importance of CXCR4/mTOR signaling axis in chondrocyte autophagy induced by SDF-1α.

## 3. Discussion

SDF-1α is the most common isoform of stromal cell-derived factor 1 [4,37]. At the same time, SDF-1α is widely distributed and plays essential roles in cell proliferation [5], migration [38], differentiation [7] and organ development [39]. Previous studies have shown that SDF-1 could potentially regulate autophagy in stem cells [40] and cancer cells [9,41,42,43]. However, these results from different cell types are not only controversial but also unclear about the actual internal mechanism. A general consensus is that SDF-1 might be a vital target for autophagy. Autophagy is a highly conserved biological process often activated under stress conditions. Through degrading and recycling intracellular components, autophagy helps cells survive in harsh environments [44]. Furthermore, cells deal with errors such as misfolded or unfolded proteins by autophagy in normal conditions. In recent years, studies have demonstrated that autophagy is related to many pathogeneses of degenerative diseases such as osteoarthritis, Alzheimer’s disease and Parkinson’s disease [45,46,47]. Therefore, the regulation of autophagy may become more clinically significant, and may provide related therapeutic targets based on autophagy.

Cartilage tissue contains only one type of cell, chondrocytes, and no blood vessels, nerves, or lymphatic vessels. Therefore, the state of chondrocytes has a significant impact on cartilage microenvironment homeostasis. OA is a degenerative joint disease [48,49]. It brings long-term chronic pain to patients and is a common cause of disability [19,50]. Until now, the pathogenesis of OA has not been fully elucidated, although many risk factors have been explored. At the same time, neither conservative treatment nor surgery can bring ideal therapeutic effects to patients. Therefore, exploring new therapeutic targets is the key to solving clinical problems. SDF-1 has been shown to promote OA progression in many pieces of research [37,51]. Moreover, the concentration of SDF-1α is also significantly increased in the synovial fluid of OA patients [4]. All of these suggest that SDF-1α might take a vital role in the progression of OA. Some studies on OA have shown that autophagy increases in the articular cartilage in early OA progression as a self-protection mechanism [52]. At the same time, SDF-1α shows the ability to regulate autophagy in other cell types. Thus, it was proposed whether SDF-1α acted as a driver for autophagy in chondrocytes. The role of SDF-1α in OA progression may perform more complex regulation.

To explore the effect of SDF-1α on chondrocytes, we performed RNA sequencing. Biological process (BP) based on Gene Ontology (GO) enrichment analysis showed that autophagy-related gene expression profiles significantly upregulated after treatment with SDF-1α. At the same time, we noticed that mitophagy-related genes were also increased. This suggests that the effect of SDF-1α on chondrocyte autophagy may involve selective autophagy, which provides directions for our future research. To confirm the RNA sequencing results, we examined the autophagy phenotype. TEM assays were performed to detect the number of autophagic vacuoles in chondrocytes with or without SDF-1α treatment. The results showed that the stimulation of SDF-1α could increase the formation of autophagic vacuoles. It is worth noting that not all autophagic vacuoles in the TEM assay exhibit the double-membrane structure. However, combined with the morphology of vesicle contents, it is believed that autophagy progression might have been in the middle and/or late stages. At this stage, autophagic vacuoles or autolysosomes contain only one limiting membrane [53,54].

To determine the changes in autophagic flux, the mRFP-GFP-LC3 adenovirus double label assays were used. Due to the faster maturation of GFP chromophore, the green-only dots occurred in some chondrocytes. In short, the results showed that SDF-1α increased chondrocyte autophagic flux. The upregulated number of yellow and free red dots indicate the increasing production of autophagosomes and autolysosomes. However, based on current research, it is still possible that substrates are not degraded after the formation of autolysosomes. Lysosomal protease inhibitors such as E64d and pepstatin should be used to examine the efficiency of the actual substrate degradation in lysosomes at different time points, detecting the changes of early and late autophagosomes [55]. Unfortunately, these are the missing pieces in this study.

In order to verify the promoting effect of SDF-1α on chondrocyte autophagy, the changes in autophagy-related protein expression were detected by western blots and immunofluorescence staining assays. Analyzing autophagy by quantifying the change of LC3B-II is one of the most widely used methods. At the same time, the guideline points out that the level of LC3B-II should compare with a “housekeeping” protein like β-actin rather than LC3B-I [56]. The results show that the essential proteins (ULK-1, Beclin1 and LC3B-II) involved in autophagy were increased, and that the protein expression of p62 was decreased. Since the autophagy process is dynamic and continuous, the time point of monitoring may have an impact on the protein level. This issue could affect the detection of autophagy levels. In order to complement and verify the alteration of autophagy-related proteins, CQ (a specific autophagy inhibitor) was used. CQ can inhibit the fusion of autophagosomes and lysosomes, thereby terminating the degradation of autophagy cargo [57]. Autophagy-related proteins will accumulate in vesicles. However, some literature shows that CQ has an inhibitory effect on CXCR4 by promoting the internalization of CXCR4 in a dose-dependent manner, thereby reducing the expression on the cell membrane [58]. It should be pointed out that at a low concentration, 20 μM, there was no significant difference in the expression of CXCR4 on the cell membrane, and the inhibitory effect on its downstream pathways such as ERK/p-ERK was also weak. Therefore, 20 μM CQ was selected to detect the accumulation of autophagy-related proteins in chondrocytes with or without SDF-1α treatment. Western blot results showed that after inhibiting autophagosome-lysosome fusion, the autophagy-related proteins were significantly increased in chondrocytes induced by SDF-1α in the presence of CQ.

There are only two receptors, CXCR4 and CXCR7, for SDF-1, and both of them are expressed on the chondrocyte membrane [29]. Although CXCR7 has a higher affinity for SDF-1 [59], most of its functions are focused on SDF-1 clearance and concentration gradient maintenance [60]. Therefore, CXCR4 was selected to be the potential receptor for testing. By using siRNA transfection targeting CXCR4, the promoting effect of SDF-1α on chondrocyte autophagy was inhibited. It was suggested that the SDF-1α/CXCR4 axis transmitted the subsequent signals.

RNA sequencing results show the alterations of autophagy-related genes in chondrocytes after treatment with SDF-1α, and that the genes related to the mTOR signaling pathway significantly changed. The mTOR signaling is a classical pathway that regulates autophagy [35]. Therefore, western blots were performed to determine the protein expression changes in the mTOR/p-mTOR signaling pathway in SDF-1α-stimulated chondrocytes. The results suggest that the phosphorylation of mTOR was inhibited. Then, the mTOR pathway activator, MHY1485, was used to authenticate the reverse. It was found that MHY1485 could partially reverse the promoting effect of SDF-1α on chondrocyte autophagy. The results also indicate that SDF-1α might have other pathways to regulate this event. Detecting the phosphorylation of mTOR is one of the methods to monitor mTOR activity. Nevertheless, further detecting the phosphorylation status of mTORC1 substrates, such as EIF4EBP1/4E-BP1/PHAS-I and RPS6KB/p70S6 kinase, could be a better approach [61,62]. On top of that, there are many upstream pathways of mTOR which are involved in regulating autophagy, such as the PI3K/AKT signaling pathway and the LKB1/AMPK signaling pathway [63,64]. Whether SDF-1α handles these signaling pathways or other pathways independent of the mTOR signaling requires more detailed research in the future. At the same time, studies have shown that the concentration of SDF-1β in the synovial fluid of OA patients was also significantly increased [4]. Whether different isoforms of SDF-1 affect chondrocyte autophagy is also worthy of exploring.

According to the analysis, the function of SDF-1α to promote chondrocyte autophagy partially explains the increase of autophagy in the early stage of OA. As OA progresses, excessive autophagy can eventually lead to cell death. SDF-1α seems to play different roles in the progression of OA. This indicates that SDF-1α could be a target for OA biological therapy in the future.

**Figure 6:**
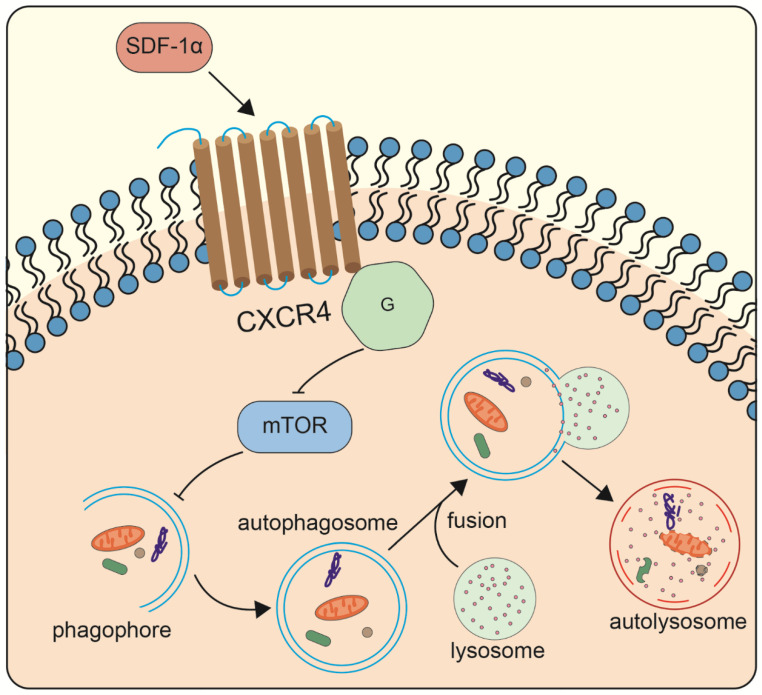
Diagrammatic sketch showing how SDF-1α mediates autophagy in chondrocytes. SDF-1 activates the subsequent cytoplasmic signals by binding to CXCR4 on the chondrocyte membrane. These signals enter the chondrocytes then inhibit the phosphorylation of the mTOR signaling, and ultimately upregulate the autophagy occurrence.

To summarize, this research demonstrates for the first time that SDF-1α promotes chondrocyte autophagy through the CXCR4/mTOR signaling axis (Figure 6). These findings may help to further understand the mechanism of OA development.

## 4. Materials and Methods

### 4.1. Cell Culture

In this study, the usage of animal samples was in compliance with ethical principles. It was reviewed and approved by the Institutional Review Board at the West China Hospital of Stomatology (No. WCHSIRB-OT-2020-048). The cells used in the experiments were all isolated from C57BL/6J mice (1–3 days old) as previously described [65,66]. Immature chondrocytes isolated from epiphyseal hyaline cartilage were extracted from knee joints. To ensure the study’s accuracy, the chondrocytes used in the experiments were at passage 1–2. The epiphyseal cartilage was cut into small pieces and then trypsinized in sufficient 0.25% protease solutions at 37 °C for 30 min. Next, the tissue was transferred to a mixture of 0.1% II collagenase and Dulbecco’s modified Eagle’s medium (DMEM, HyClone) (1:1.5) for 8–12 h at 37 °C. Then, an equal volume of media containing 10% heat-inactivated fetal bovine serum (FBS, HyClone, Logan, UT, USA) and 1% penicillin-streptomycin (HyClone) was added. Cell suspension was centrifuged at 1000 rpm for 5 min and replaced with fresh 10% FBS DMEM containing 1% penicillin–streptomycin solution. Cells were seeded on plates and incubated at 37 °C in a humidified atmosphere of 5% CO_2_.

### 4.2. Transmission Electron Microscope (TEM)

Isolated chondrocytes at P1 were seeded onto 60 mm cell culture dishes at 70% confluency. After incubating with or without SDF-1α (#250-20A, Pepro-tech, Cranbury, NJ, USA) for 24 h, cells were trypsinized in 0.25% protease solutions. Cell samples were collected by centrifuging at 1000 rpm for 5 min and removing the supernatant. After adding 0.5% glutaraldehyde, cells were resuspended at 4 °C for 10 min and then centrifuged at 12,000 rpm for 10 min. The cells were collected and 3% glutaraldehyde was slowly added. The cell samples were kept at a low temperature and transported to Chengdu Lilai Biotechnology Co., Ltd. (Chengdu, China) for TEM (JEM-1400FLASH; JEOL, Tokyo, Japan).

### 4.3. mRFP-GFP-LC3 Adenovirus Double Label Assay

Isolated chondrocytes at P1 were seeded in 24-well plates at 50% confluency. mRFP-GFP-LC3 adenovirus (MOI = 70; Hanbio, Shanghai, China) was transfected in 50% volume of culture media for 4 h. Supplemented with an equal volume of media, and cells were transfected for 4 h. Then, the culture media was changed to incubate chondrocytes. For the confocal laser microscope (CLSM), cells were seeded in a specialized dish. At room temperature (RT), cells were fixed with 4% paraformaldehyde (PFA) for 10–15 min. The nuclei were stained with Dapi (D9542, Sigma, St. Louis, MO, USA) for 10 min. Finally, the CLSM (FV3000; Olympus, Tokyo, Japan) was used to obtain the images.

### 4.4. Western Blot Analysis

With the fresh culture media containing 10% FBS DMEM, isolated chondrocytes at P1 were seeded in 6-well plates at 70% confluency. The treatments used in the experiments included SDF-1α for 6 or 48 h (50, 100 and 200 ng/mL); chloroquine (CQ, C6628, Sigma MO, USA) for 3 h at 20 μM; MHY1485 (SML0810, Sigma, St. Louis, MO, USA) for 2 h at 100 nM. Chondrocytes were lysed in an ice bath with the mixture of PMSF (P7626, Sigma, St. Louis, MO, USA) protease inhibitor and RIPA lysis buffer (P0013B, Beyotime, Shanghai, China) (1:100) to collect protein samples. The Beyotime BCA kit (P0010, Beyotime, Shanghai, China) was used to confirm the concentration of protein samples. Subsequently, the Bio-Rad Laemmli sample buffer (Bio-Rad Laboratories, Hercules, CA, USA) was added (1:1) for denaturation at 100 °C for 5–8 min. Through 10% or 15% SDS-PAGE, the protein samples were separated. It was then transferred onto a polyvinylidene difluoride (PVDF) membrane (Millipore, Billerica, MA, USA). The 5% skim milk containing 0.05% Tween 20 was used for blockage for 1 h. The primary antibodies including ULK-1 (anti-rabbit, 381887, zen-bio, Chengdu, China), p62 (anti-rabbit, 380612, zen-bio, Chengdu, China), Beclin-1 (anti-rabbit, R22856, zen-bio, Chengdu, China), LC3B (anti-rabbit, 382687, zen-bio, Chengdu, China), mTOR (anti-rabbit, 380411, zen-bio, Chengdu, China), p-mTOR (anti-rabbit, 381557, zen-bio, Chengdu, China), Collagen II (anti-rabbit, ab34712, Abcam, Cambridge, UK), Aggrecan (anti-mouse, ab3778, Abcam, Cambridge, UK), β-actin (anti-mouse, sc-47778, Santa Cruz Biotechnology, Santa Cruz, CA, USA) were incubated with PVDF membranes for 8–12 h at 4 °C. The corresponding secondary antibodies were used for the conjugation reaction for 2 h at RT. Finally, in order to visualize the protein bands, the Immobilon^®^ Western (P90719, Millipore, Billerica, MA, USA) kit was used. The results were normalized by the expression of β-actin and analyzed by Image J software (v2.9.0, NIH, Bethesda, MD, USA).

### 4.5. Immunofluorescence and Confocal Laser Scanning Microscope (CLSM)

In order to observe immunofluorescent images by CLSM, isolated chondrocytes at P1 were cultured onto special dishes for 48 h, as previously described [67,68]. Cells were rinsed three times with warm 1 × PBS buffer after discarding the culture media and then fixed with 4% cold PFA for 10–15 min. 0.25% Triton X-100 (Beyotime, China, Shanghai) was used to permeabilize chondrocytes for 10 min. Then, samples were blocked with 5% bovine serum albumin for 1 h. The primary antibodies related to autophagy (ULK-1, p62, Beclin-1 and LC3B) were added and incubated at 4 °C for 8–12 h. After that, the secondary antibody conjugated to Alexa Fluor 647 (1:300 dilution, ab150075, Abcam, Cambridge, UK) was added to the cells and incubated at RT for 2 h in the dark. Cytoskeleton was stained with phalloidin (Invitrogen, Carlsbad, CA, USA) overnight at 4 °C. Nuclei were stained with Dapi (D9542, Sigma, St. Louis, MO, USA) for 10 min at RT. Finally, immunofluorescence images were obtained by CLSM (FV3000; Olympus, Tokyo, Japan). Image J software (v2.9.0, NIH, Bethesda, MD, USA) was used to analyze the results.

### 4.6. RNA Sequencing

With or without 200 ng/mL SDF-1α treatment for 48 h, cell lysates of chondrocytes at P1 were collected using Trizol (Thermo Fisher Scientific, Waltham, MA, USA). After confirming the integrity of the collected mRNA samples by the RNA Nano 6000 Assay Kit of the Bioanalyzer 2100 system (Agilent Technologies, Palo Alto, CA, USA), the total mRNA was sent to Shanghai Lifegenes Biotechnology Co., Ltd. (Shanghai, China) for transcriptome analysis. In accordance with the manufacturer’s instructions, each sample used 1.5 μg of RNA as the input material for RNA detection. The index-coded samples were clustered using a HiSeq 4000 PE Cluster Kit (Illumina, San Diego, CA, USA). To obtain clean data, the raw data were first processed with internal scripts. Subsequently, HISAT2 v2.1.0 was used to match the reference genome, collecting the data. Data analysis using HTSeq v0.6.1. Via Kyoto Encyclopaedia of Genes and Genomes enrichment analysis/Gene Ontology enrichment analysis screened out differential genomes. Significance was set at *p* < 0.05 and |Foldchange| ≥ 1.5.

### 4.7. Small Interfering RNA (siRNA) Transfection

Chondrocytes at P1 were seeded in 6-well plates overnight. Then, the siRNA oligonucleotides targeting CXCR4 (Hanbio, Shanghai, China) were transiently transfected with Lipofectamine RNAiMAX (Invitrogen, Burlington, ON, Canada). The siRNA sequences used in subsequent experiments are listed as follows: sense, 5′-GCCUCAAGAUCCUUUCCAATT-3′; antisense, 5′- UUGGAAAGGAUCUUGAGGCTT -3′. The siRNA negative control group was set as the si-NC group in the experiments. After transfection for 24 h, protein samples were extracted for the western blot assay to assess the efficiency of CXCR4 knockdown.

### 4.8. Quantitative Real-Time Polymerase Chain Reaction (qPCR)

The total RNA was extracted from chondrocytes at P1 treated with or without 200 ng/mL SDF-1α for 24 h by RNeasy Plus Mini Kit (74136, Qiagen, Valencia, CA, USA). The extracted RNA samples were quantitated with the Nanodrop spectrophotometer (Thermo Fisher Scientific, Waltham, MA, USA). Then, the total RNA was reversely transcripted into cDNA by the cDNA synthesis kit (K1621-RevertAid, Thermo Fisher Scientific, Mbi, MD, USA). Primers designed for qPCR are listed in Table S5. Following the operating procedure, qPCR was performed with the iCycler (Bio-Rad, Hercules, CA, USA). The expression of genes was normalized by the expression of the glyceraldehyde 3-phosphate dehydrogenase (GAPDH) and calculated by a ΔΔCt method.

### 4.9. Statistical Analysis

Experiments were independently repeated at least in triplicate (*n* ≥ 3). Specifically, we randomly analyzed cells (12–15 cell for immunofluorescence, 3–5 cells for mRFP-GFP-LC3 adenovirus double label assay and 3 cells for TEM assay) per group from three independent experiments, and obtained the three average data from three independent experiments, respectively. We then made statistical difference analysis about these average data between control group and SDF-1α group. Differences among groups were statistically analyzed by one-way analysis of variance and Student *t*-test. Data are presented as the mean ± standard deviation (SD) and plotted with GraphPad Prism 8 software. Only with *p* value < 0.05 is the difference considered to be statistically significant.

## Data Availability

Any data generated in this study are available from the corresponding author upon request in addition to source data.

## Supplementary Materials

The following supporting information can be downloaded at: https://www.mdpi.com/article/10.3390/ijms24021710/s1. Figure S1: The protein expressions of autophagy-related proteins shown in Figure 2A. Figure S2: The protein expressions of autophagy-related proteins shown in Figure 2D. Figure S3: Determination of receptor (CXCR4) expressions by different small interfering RNA (siRNA) transfection. Figure S4: The protein expressions of autophagy-related proteins shown in Figure 3D. Figure S5: Quantitative analysis of mTOR signaling shown in Figure 5A. Figure S6: The protein expressions of autophagy-related proteins shown in Figure 5E. Figure S7: the protein expressions of autophagy-related proteins shown in Figure 5F. Figure S8: Quantitative analysis of the protein expressions of autophagy-related proteins shown in Figure 5E. The results were based on three independent experiments (*n* = 3). All significance data presented were based on two-tailed Student’s *t*-tests. Figure S9: Quantitative analysis of the protein expressions of autophagy-related proteins shown in Figure 5F. The results were based on three independent experiments (*n* = 3). All significance data presented were based on two-tailed Student’s *t*-tests.Table S1: Autophagy-related biological process (BP) based on GO analysis. Table S2: RNA sequencing-based expression of all chemokine (C-X-C motif) receptors in chondrocytes. Table S3: RNA sequencing-based candidates in autophagy-related proteins and signaling. Table S4: RNA sequencing-based gene candidates in chondrocyte phenotype. Table S5: Primers designed for qPCR. Supplementary figures, tables and original images of western blots are all attached as a Supplementary Information File.
