# Supplementary material for: SDF-1α Promotes Chondrocyte Autophagy through CXCR4/mTOR Signaling Axis
Source: Int J Mol Sci. 2023 Jan 15;24(2):1710. doi: 10.3390/ijms24021710 (PMC9867011; doi:10.3390/ijms24021710)

Supplementary Materials for Original Paper:

SDF-1α promotes chondrocyte autophagy through CXCR4/mTOR signaling axis

Jiazhou Li, Hao Chen, Lang Cai, Daimo Guo, Demao Zhang, Xuedong Zhou, Jing Xie

1. Supplementary Figures

FigureS1

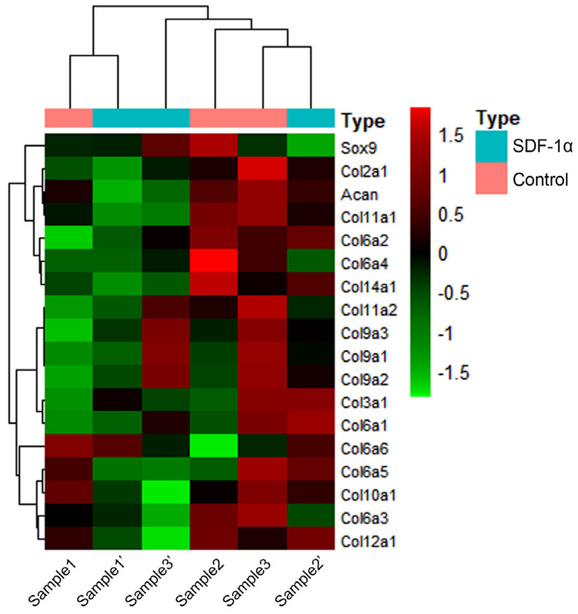

Figure S1. SDF-1α did not significantly change chondrocyte phenotype after treatment for 48 h. Pheatmap did not isolate and cluster the genes of chondrocyte phenotype between control and SDF-1α group. Chondrocytes specifically express Sox9, and secrete aggrecan and tissue-specific collagens including types II, III, VI, IX, X, XI, XII and XIV. All gene subtypes involving protein markers of chondrocyte phenotype were listed based on the RNA sequencing. The specific data generated from RNA sequencing (FPKM) were listed in table S4.

Figure S2

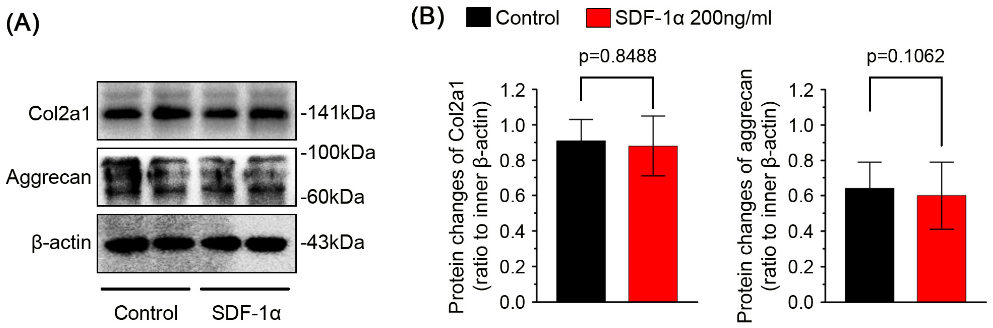

Figure S2. SDF-1α did not significantly change the expressions of collagen type II and aggrecan in chondrocyte after treatment for 48 h. The results were based on three independent experiments (n = 3). All significance data presented were based on two-tailed Student's t-tests.

Figure S3

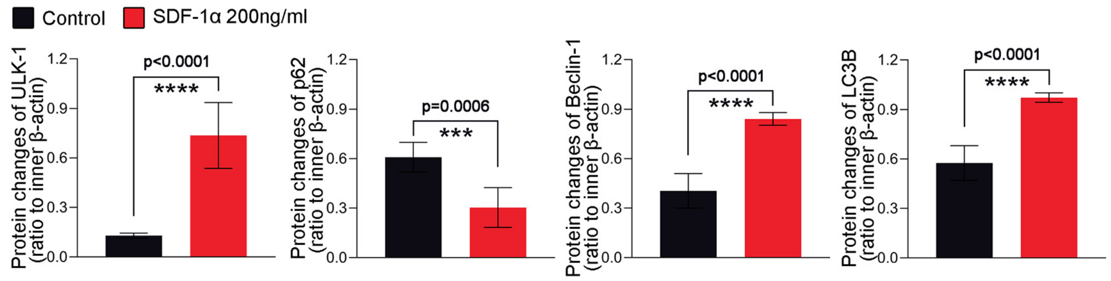

Figure S3. Quantitative analysis of the protein expressions of autophagy-related proteins shown in Figure 2A. The results were based on three independent experiments (n = 3). All significance data presented were based on two-tailed Student's t tests.

Figure S4

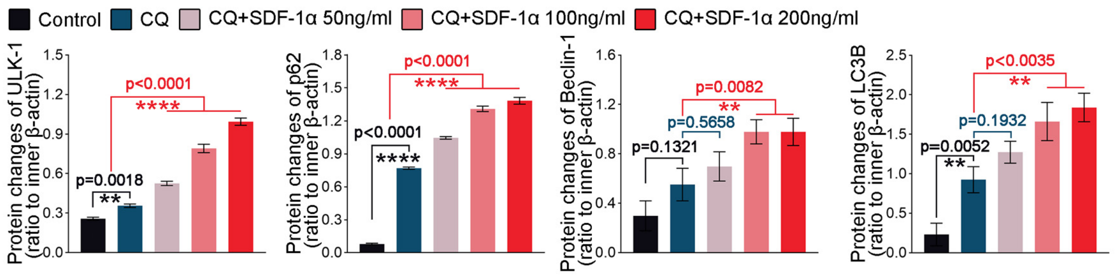

Figure S4. Quantitative analysis of the protein expressions of autophagy-related proteins shown in Figure 2D. The results were based on three independent experiments (n = 3). All significance data presented were based on two-tailed Student's t-tests.

Figure S5

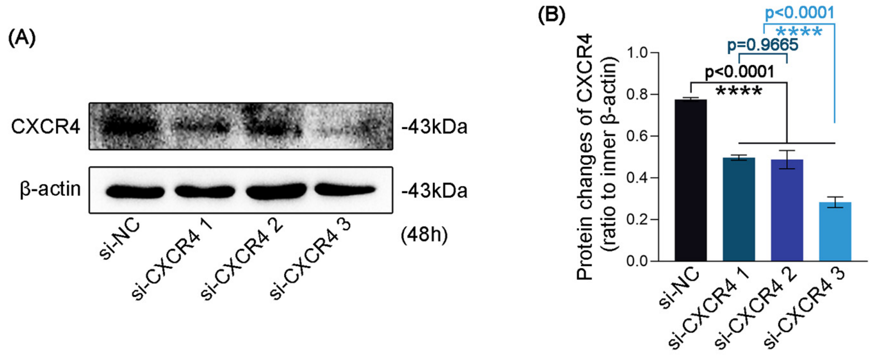

Figure S5. Determination of receptor (CXCR4) expressions by different small interfering RNA (siRNA) transfection.

(A). Representative western blots showing the expression changes of CXCR4 in chondrocytes by siRNA transfection. The results showed that si-CXCR4 (50 nM) inhibited CXCR4 expression successfully. The gel images were chosen based on three independent experiments (n = 3).

(B). Quantitative analysis of CXCR4 expression shown in (A). The results were based on three independent experiments (n=3). All significance data presented were based on two-tailed Student's t-tests.

Figure S6

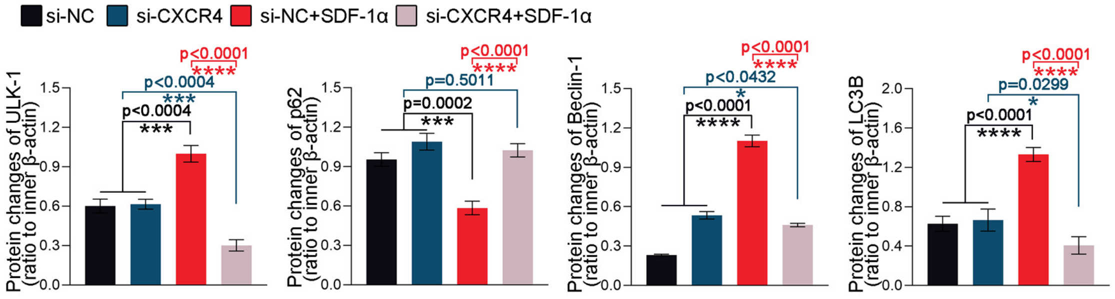

Figure S6. Quantitative analysis of the protein expressions of autophagy-related proteins shown in Figure 3D. The results were based on three independent experiments (n = 3). All significance data presented were based on two-tailed Student's t-tests.

Figure S7

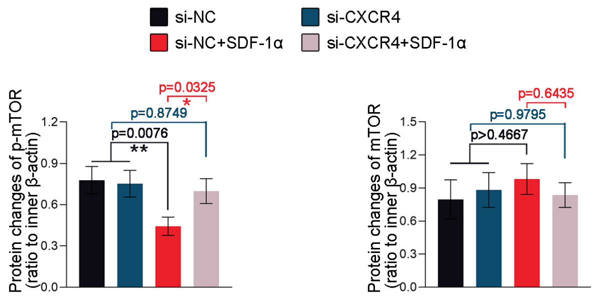

Figure S7. Quantitative analysis of mTOR signaling shown in Figure 5A. The results were based on three independent experiments (n = 3). All significance data presented were based on two-tailed Student's t-tests.

Figure S8

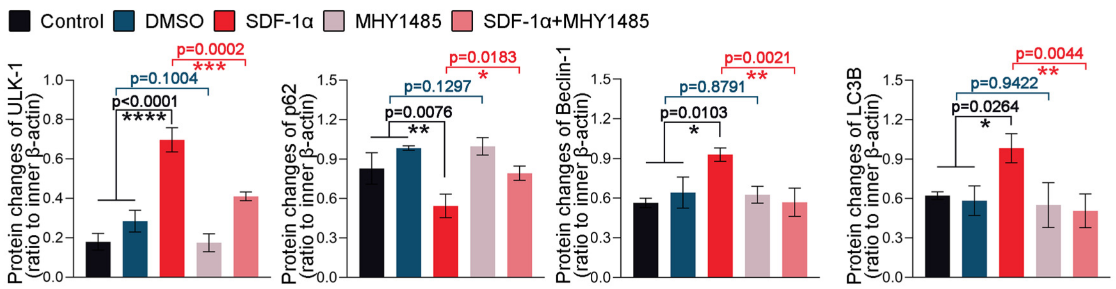

Figure S8. Quantitative analysis of the protein expressions of autophagy-related proteins shown in Figure 5E. The results were based on three independent experiments (n = 3). All significance data presented were based on two-tailed Student's t-tests.

Figure S9

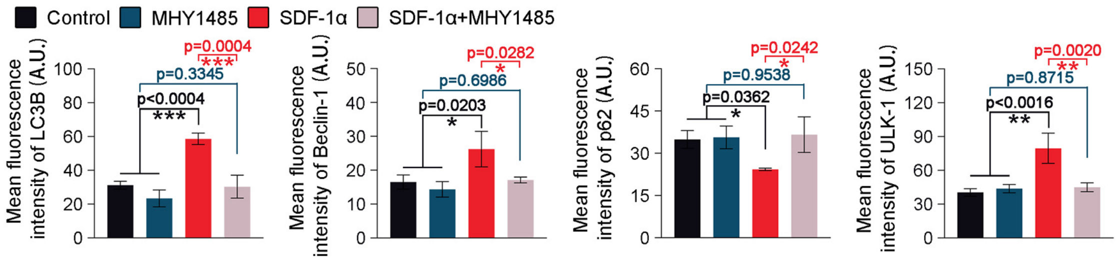

Figure S9. Quantitative analysis of the protein expressions of autophagy-related proteins shown in Figure 5F. The results were based on three independent experiments (n = 3). All significance data presented were based on two-tailed Student's t-tests.

2. Supplementary tables

Table S1. Autophagy-related biological process (BP) based on GO analysis.

Upregulated GO analysis (Biological Processes)						
ONTOLOGY	ID	Description	GeneRatio	BgRatio	p.adjust	geneID
BP	GO:0034660	ncRNA metabolic process	3/27	451/21602	0.0182348	Hsd17b10/2210016F16Rik/Pih1d1
BP	GO:0000045	autophagosome assembly	4/67	298/21602	0.0002101	Prkaa2/Ulk1/Becn1/Sqstm1
BP	GO:0000423	mitophagy	3/67	216/21602	0.0002021	Ulk1/beclin1/Sqstm1
BP	GO:0048732	gland development	3/27	469/21602	0.0202093	Clcn2/Cyp7b1/Usf2
BP	GO:0007005	mitochondrion organization	3/27	490/21602	0.0226579	Uqcc3/Uqcr10/Hsd17b10
BP	GO:0017062	respiratory chain complex III assembly	2/27	10/21602	6.728E-05	Uqcc3/Uqcr10
BP	GO:0034551	mitochondrial respiratory chain complex III assembly	2/27	10/21602	6.728E-05	Uqcc3/Uqcr10
BP	GO:0006122	mitochondrial electron transport, ubiquinol to cytochro	2/27	12/21602	9.853E-05	Uqcc3/Uqcr10
BP	GO:0017004	cytochrome complex assembly	2/27	36/21602	0.0009233	Uqcc3/Uqcr10
BP	GO:0042775	mitochondrial ATP synthesis coupled electron transpo	2/27	59/21602	0.0024635	Uqcc3/Uqcr10
BP	GO:0042773	ATP synthesis coupled electron transport	2/27	63/21602	0.0028032	Uqcc3/Uqcr10
BP	GO:0006400	tRNA modification	2/27	75/21602	0.0039465	Hsd17b10/2210016F16Rik
BP	GO:0022904	respiratory electron transport chain	2/27	82/21602	0.0046977	Uqcc3/Uqcr10
BP	GO:0033108	mitochondrial respiratory chain complex assembly	2/27	84/21602	0.0049235	Uqcc3/Uqcr10
BP	GO:0022900	electron transport chain	2/27	85/21602	0.0050383	Uqcc3/Uqcr10

Downregulated GO analysis (Biological Processes)						
ONTOLOGY	ID	Description	GeneRatio	BgRatio	p.adjust	geneID
BP	GO:0072521	purine-containing compound metabolic process	6/39	402/21602	7.763E-05	Prkaa2/Gmpr2/Pemt/Zbtb20/Nos1/Pd
BP	GO:0006163	purine nucleotide metabolic process	5/39	357/21602	0.0004349	Prkaa2/Gmpr2/Zbtb20/Nos1/Pde5a
BP	GO:0009117	nucleotide metabolic process	5/39	431/21602	0.0010171	Prkaa2/Gmpr2/Zbtb20/Nos1/Pde5a
BP	GO:0006753	nucleoside phosphate metabolic process	5/39	439/21602	0.0011039	Prkaa2/Gmpr2/Zbtb20/Nos1/Pde5a
BP	GO:0006790	sulfur compound metabolic process	4/39	285/21602	0.0016965	Chsy1/Xylt1/Gstt1/Pemt
BP	GO:0009150	purine ribonucleotide metabolic process	4/39	325/21602	0.0027318	Prkaa2/Gmpr2/Zbtb20/Pde5a
BP	GO:0009259	ribonucleotide metabolic process	4/39	335/21602	0.003046	Prkaa2/Gmpr2/Zbtb20/Pde5a
BP	GO:0019693	ribose phosphate metabolic process	4/39	346/21602	0.0034194	Prkaa2/Gmpr2/Zbtb20/Pde5a
BP	GO:0018205	peptidyl-lysine modification	4/39	368/21602	0.0042578	Kmt2c/Prkaa2/Atat1/Nos1
BP	GO:0001503	ossification	4/39	401/21602	0.0057611	Chsy1/Dchs1/Xylt1/Cdk6
BP	GO:0031331	positive regulation of cellular catabolic process	4/39	402/21602	0.0058115	Abca2/Prkaa2/Zbtb20/Sh3rf2
BP	GO:0009896	positive regulation of catabolic process	4/39	469/21602	0.0098991	Abca2/Prkaa2/Zbtb20/Sh3rf2
BP	GO:0045981	positive regulation of nucleotide metabolic process	3/39	42/21602	5.948E-05	Prkaa2/Zbtb20/Nos1
BP	GO:1900544	positive regulation of purine nucleotide metabolic proc	3/39	42/21602	5.948E-05	Prkaa2/Zbtb20/Nos1
BP	GO:0043279	response to alkaloid	3/39	75/21602	0.0003358	Prkaa2/Casp7/Nos1

Table S2. RNA sequencing-based expression of all chemokine (C-X-C motif) receptors in chondrocytes

ensembl_id	gene_name	gene_biotype	description	Sample-1_FPK	Sample-2_FPK	Sample-3_FPK
ENSMUSG000000484	Cxcr1	protein_codin	chemokine (C-X-C motif) receptor	0.363205206	0.606732728	0.425124251
ENSMUSG000000261	Cxcr2	protein_codin	chemokine (C-X-C motif) receptor	0	0.019786494	0.014021327
ENSMUSG000000502	Cxcr3	protein_codin	chemokine (C-X-C motif) receptor	0	0	0.002141459
ENSMUSG000000453	Cxcr4	protein_codin	chemokine (C-X-C motif) receptor	0.269571386	0.366925644	0.312451625
ENSMUSG000000478	Cxcr5	protein_codin	chemokine (C-X-C motif) receptor	0.09751602	0.01810001	0.041254153
ENSMUSG000000485	Cxcr6	protein_codin	chemokine (C-X-C motif) receptor	0.056088966	0.093696375	0.064414959
ENSMUSG000000443	Cxcr7	protein_codin	chemokine (C-X-C motif) receptor	0	0	0.001241254

"0" means not detectable

Table S3. RNA sequencing-based candidates in autophagy-related proteins and signaling

ensembl_id	gene_na	gene_biotype	description	log2Fold	p.adj	control-1_F	control-2_F	control-3_F	test-1_FPKM	test-2_FPKM	test-3_FPKM
ENSMUSG000000322	Crabp1	protein_coding	cellular retinoic a	0.92198	0.00837	2.4308482	2.4063534	2.2021215	4.0684567	4.3368569	5.2235428
ENSMUSG000000228	Clcn2	protein_coding	chloride channel,	0.437	0.01431	1.4747989	1.592356	1.523697	2.0809426	2.011457	2.1068644
ENSMUSG000000789	Sec61g	protein_coding	SEC61, gamma s	0.28485	0.01448	21.538481	20.045967	21.022133	25.217942	24.232959	25.940123
ENSMUSG000000034	Pih1d1	protein_coding	PIH1 domain cor	0.26596	0.0405	17.774274	18.24205	18.051233	20.928646	21.259875	22.812918
ENSMUSG000000539	Pde5a	protein_coding	phosphodiesteras	-0.294	0.04863	2.0656811	1.9630714	1.9523233	1.6835473	1.569875	1.6314681
ENSMUSG000000285	Prkaa2	protein_coding	protein kinase, A	-0.4243	0.01999	1.8144172	1.9979467	1.7259469	1.4657102	1.5444651	1.3968527
ENSMUSG000000263	Tfcp2l1	protein_coding	transcription fact	-0.6189	0.04603	1.1360957	0.9521165	1.073699	0.7665958	0.7265946	0.6024002
ENSMUSG000000623	Dgkk	protein_coding	diacylglycerol kin	-1.5106	0.04817	0.0983516	0.0926797	0.0914979	0.0198191	0.0435696	0.050165
ENSMUSG000000262	Htr2b	protein_coding	5-hydroxytryptam	-3.6901	0.03515	0.2287119	0.0849027	0.1526613	0.0221907	0.0698745	0
ENSMUSG000000295	Ulk1	protein_coding	unc-51 like kinas	0.86715	0.0007	13.002863	13.542697	13.983949	15.00428	15.002944	14.256968
ENSMUSG000000350	Becn1	protein_coding	beclin 1, autopha	0.67365	0.00665	5.3814444	5.5632492	5.3874895	6.6094345	6.915703	7.5687984
ENSMUSG000000158	Sqstm1	protein_coding	sequestosome 1	0.88723	0.00282	187.81204	187.80206	178.65948	204.83672	209.54987	208.62592

Table S4. RNA sequencing-based gene candidates in chondrocyte phenotype

ensembl_id	gene_name	gene_biotype	description	log2FoldChange	p.adj	control-1_FPKM	control-2_FPKM	control-2_FPKM	test-1_FPKM	test-2_FPKM	test-3_FPKM
ENSMUSG00000030607	Acan	protein_coding	aggrecan	-0.061478157	0.060889247	186.3810494	192.2545258	203.447776	162.5623168	189.2358363	172.6176523
ENSMUSG00000000567	Sox9	protein_coding	SRY (sex determining regi	0.000889864	0.906775637	58.37095906	61.25112488	58.29649456	58.47298318	56.46545475	59.87582228
ENSMUSG00000022483	Col2a1	protein_coding	collagen, type II, alpha 1	-0.016513971	0.437178281	2723.590034	2825.232254	3055.935334	2623.431384	2829.456459	2776.725396
ENSMUSG00000026043	Col3a1	protein_coding	collagen, type III, alpha 1	0.022479999	0.850445298	686.048657	701.2122169	751.8194719	721.7267836	752.1012147	706.9930436
ENSMUSG00000001119	Col6a1	protein_coding	collagen, type VI, alpha 1	0.00776187	0.80641792	429.6332744	435.2114797	449.0284026	433.9847849	452.3978546	442.0197332
ENSMUSG00000020241	Col6a2	protein_coding	collagen, type VI, alpha 2	0.016098732	0.986743648	145.4402203	159.3969855	155.9666597	150.5664787	157.8219857	153.9946513
ENSMUSG00000048126	Col6a3	protein_coding	collagen, type VI, alpha 3	-0.050934117	0.306386926	183.850735	191.2359886	195.9004006	181.5839667	179.3352565	170.8159804
ENSMUSG00000032572	Col6a4	protein_coding	collagen, type VI, alpha 4	0	0.693546356	0	0.032235147	0.014147134	0	0.001259759	0.007019338
ENSMUSG00000091345	Col6a5	protein_coding	collagen, type VI, alpha 5	-0.350706567	0.041277586	0.040271675	0.018958965	0.057663136	0.015071212	0.045225685	0.012715792
ENSMUSG00000043719	Col6a6	protein_coding	collagen, type VI, alpha 6	-0.160489408	0.789509658	0.407660795	0.038895354	0.223708698	0.34050152	0.324522541	0.228522873
ENSMUSG00000026147	Col9a1	protein_coding	collagen, type IX, alpha 1	0.015671133	0.972933602	164.4171275	171.2393486	186.9848029	168.7193257	174.2491385	184.6420185
ENSMUSG00000028626	Col9a2	protein_coding	collagen, type IX, alpha 2	0.038921697	0.94404959	68.06418627	74.21858715	87.35582952	74.16416152	78.39687448	84.82980422
ENSMUSG00000027570	Col9a3	protein_coding	collagen, type IX, alpha 3	0.056649727	0.839195704	81.5470818	93.25368575	106.2337829	92.04860832	95.26422322	105.1397618
ENSMUSG00000039462	Col10a1	protein_coding	collagen, type X, alpha 1	-0.035619187	0.211784592	7.318561701	7.012571486	7.476032739	6.798657276	7.123552475	6.21413247
ENSMUSG00000027966	Col11a1	protein_coding	collagen, type XI, alpha 1	-0.016205887	0.190610914	374.4879607	388.5474858	393.3208802	360.4407595	378.3694256	363.0118758
ENSMUSG00000024330	Col11a2	protein_coding	collagen, type XI, alpha 2	0.02801534	0.875330901	223.4413068	254.9887543	285.5837748	237.2106031	245.3654759	262.5868191
ENSMUSG00000032332	Col12a1	protein_coding	collagen, type XII, alpha 1	-0.029169821	0.188698662	152.874159	158.7887546	151.7433852	144.048384	159.3598546	132.6472125
ENSMUSG00000022371	Col14a1	protein_coding	collagen, type XIV, alpha 1	-0.05708115	0.186803028	10.85703382	16.15878457	12.07388612	9.312039556	13.25633657	10.47085649

Note: all data between control group and SDF-1α group were shown to be no significant difference.

Table S5. Primers designed for qPCR.

Genes	Forward sequence	Reverse sequence	Product size (bp)
**Gapdh**	TCAAGCTCATTTCCTGGTATGAC	GGGATAGGGCCTCTCTTGCT	141
**Prkaa2**	GGCAAAGTGAAGACTACCAGG	CTTCAACCCGCCCATGTTTG	114
**Pih1d1**	GAAGGCCGCAGTGGATGAC	GGTGCCAAAAACGTCGAGTC	146
**Ulk1**	ATTACCAGCGCATCGAGCAA	TGGGGAGAAGGTGTGTAGGG	199
**Becn1**	AGGCATGGAGGGGTCTAAGG	AATGGCTCCTGTGAGTTCCTG	139
**Sqstm1**	GATAGCCTTGGAGTCGGTGG	TCAGCCTCTGTAGATGGGTCC	120

Gapdh, glyceraldehyde-3-phosphate dehydrogenase; Prkaa2, 5'-AMP-activated protein kinase catalytic subunit alpha-2; Pih1d1, PIH1 domain containing 1; Ulk1, unc-51 like kinase 1; Becn1, beclin 1, autophagy related; Sqstm1, sequestosome 1.

3. Original images about western blots in this article

Figure 2A control—control--SDF-1α-- SDF-1α

Figure 2A β-actin

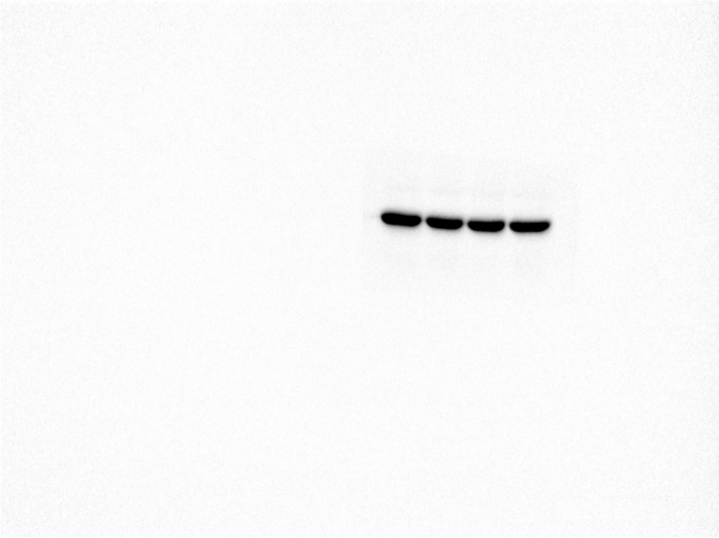

Figure 2A ULK-1

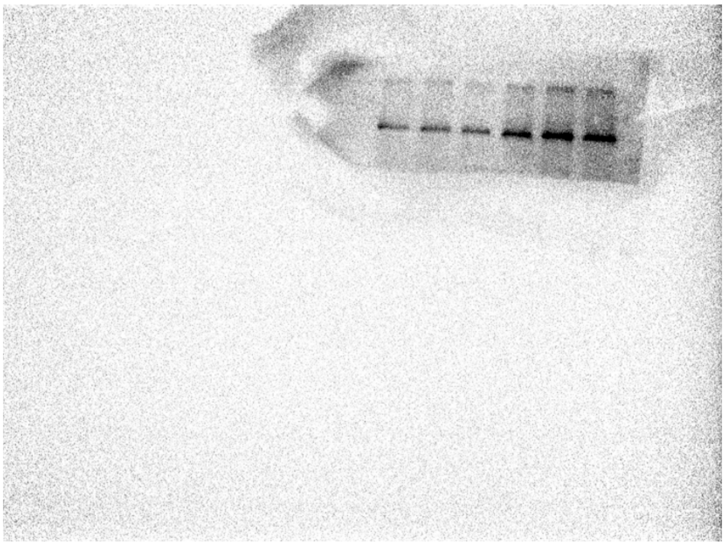

Figure 2A p62

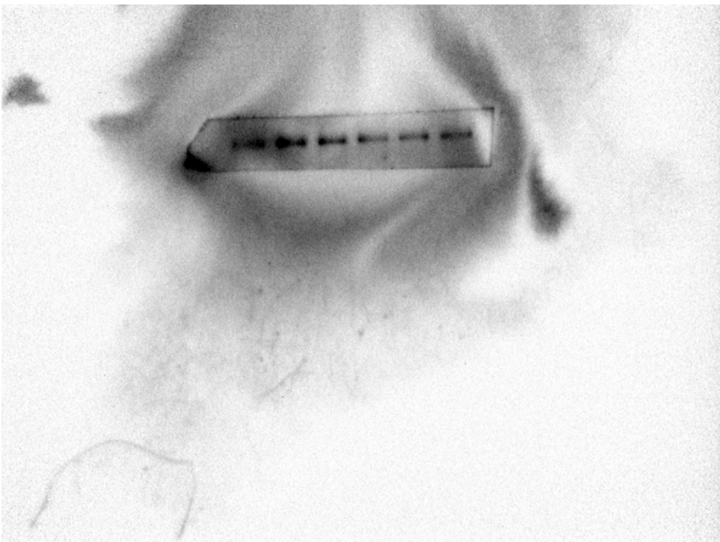

Figure 2A Beclin-1

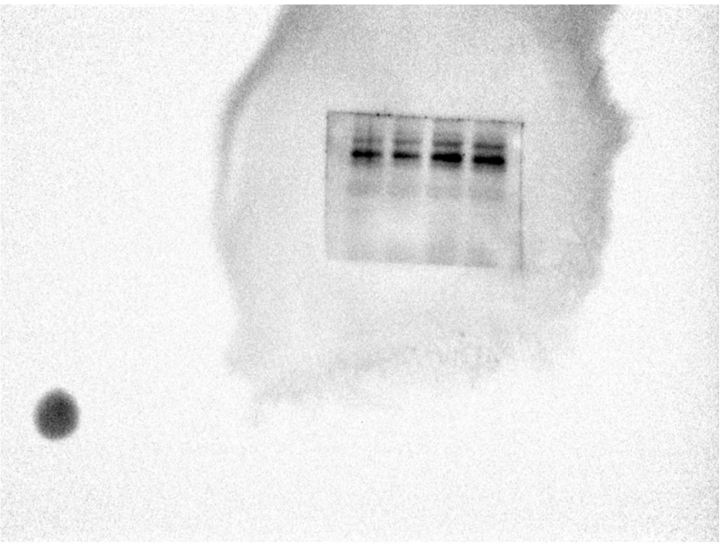

Figure 2A LC3B

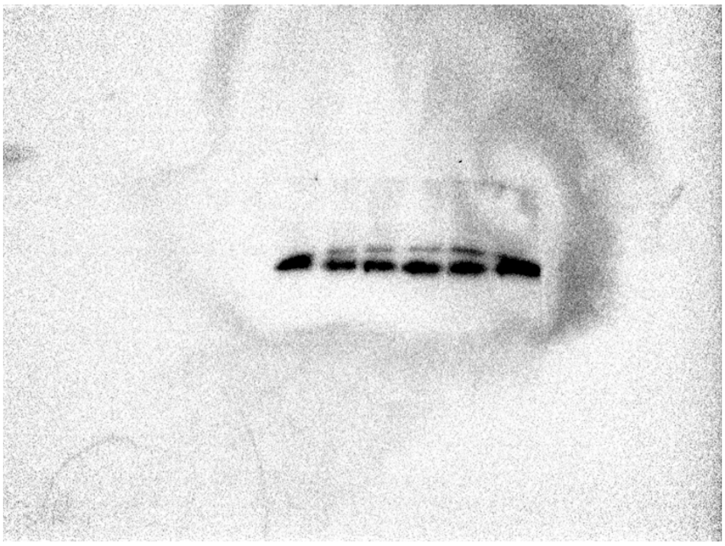

Figure 2B control--CQ--CQ+SDF-1α(50--100--200 μg/ml)

Figure 2B β-actin

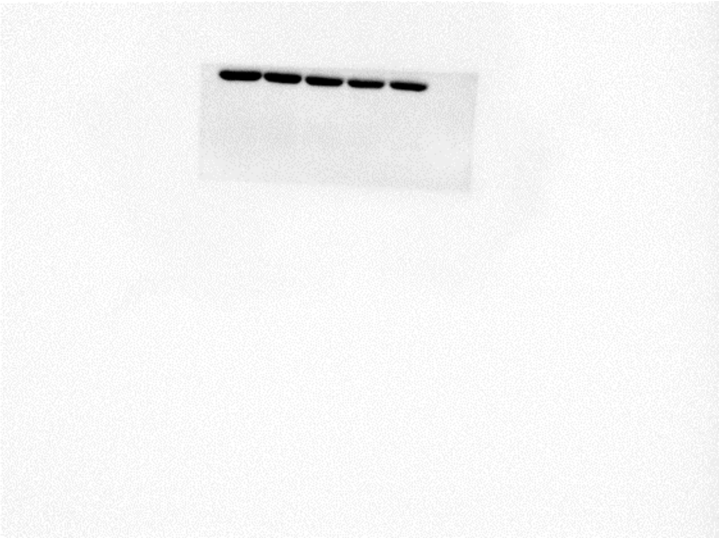

Figure 2B ULK-1

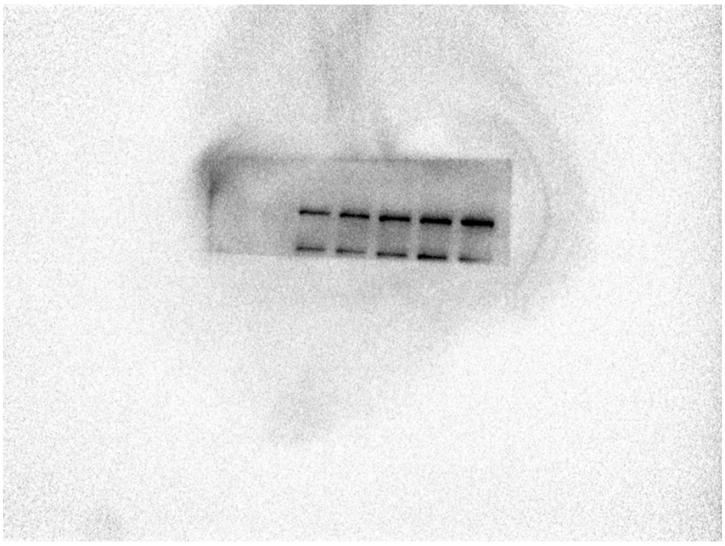

Figure 2B p62

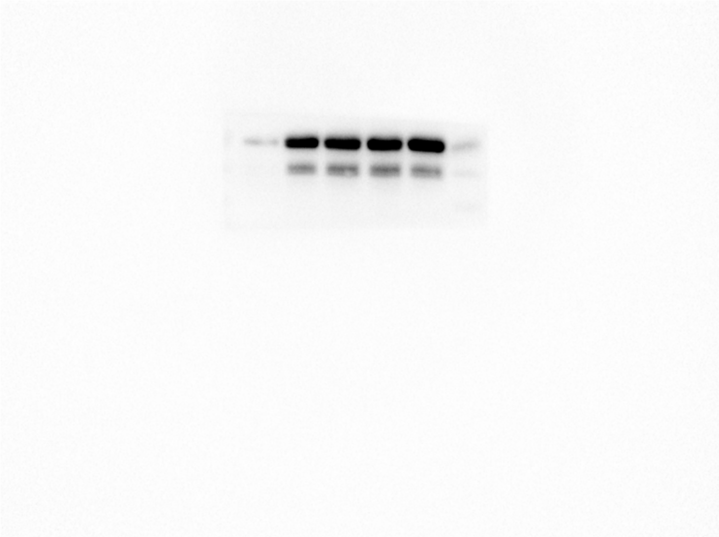

Figure 2B Beclin-1

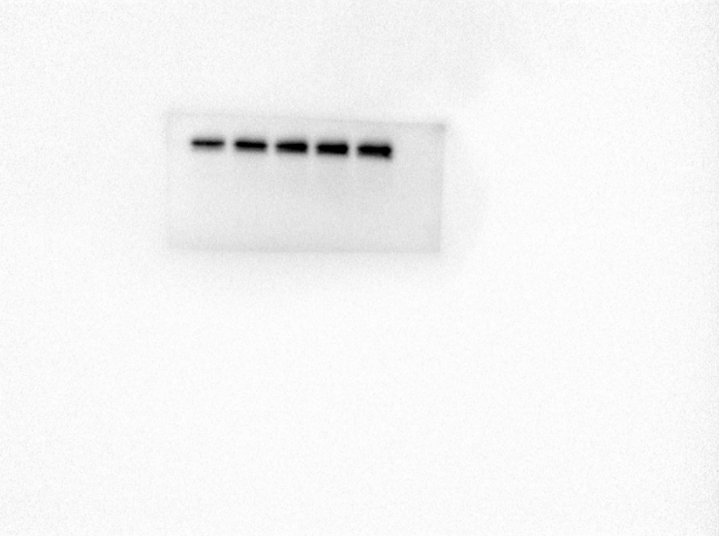

Figure 2B LC3B

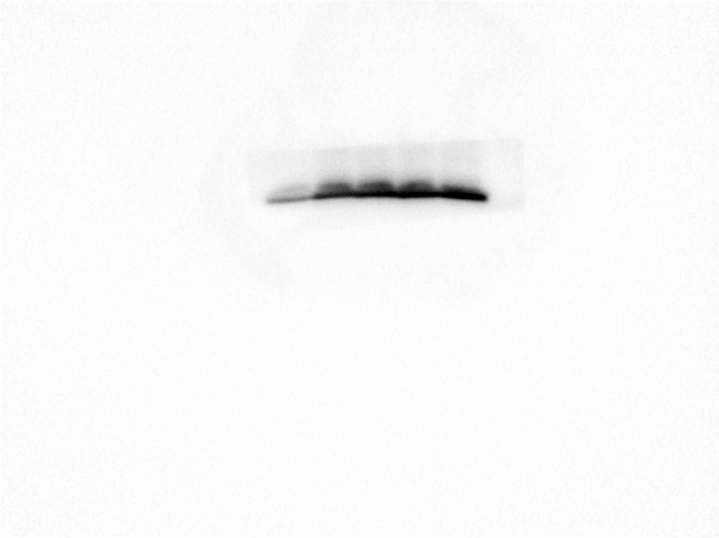

Figure 3D si-NC--si-CXCR4--si-NC+ SDF-1α--si-CXCR4+ SDF-1α

Figure 3D β-actin

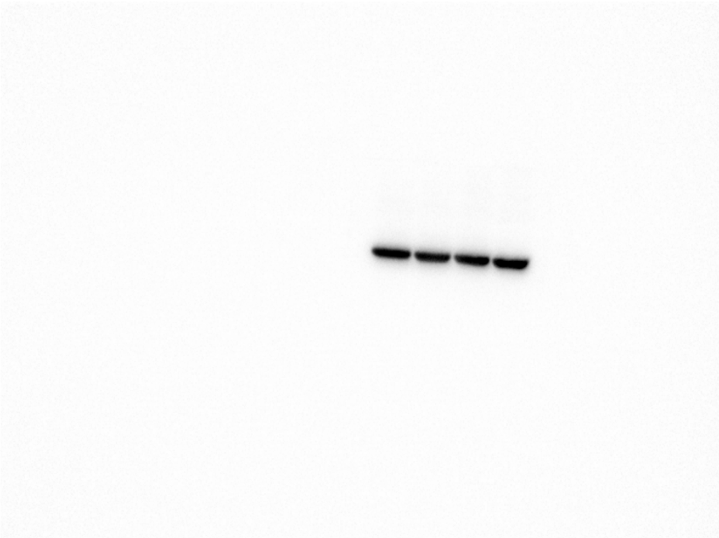

Figure 3D ULK-1

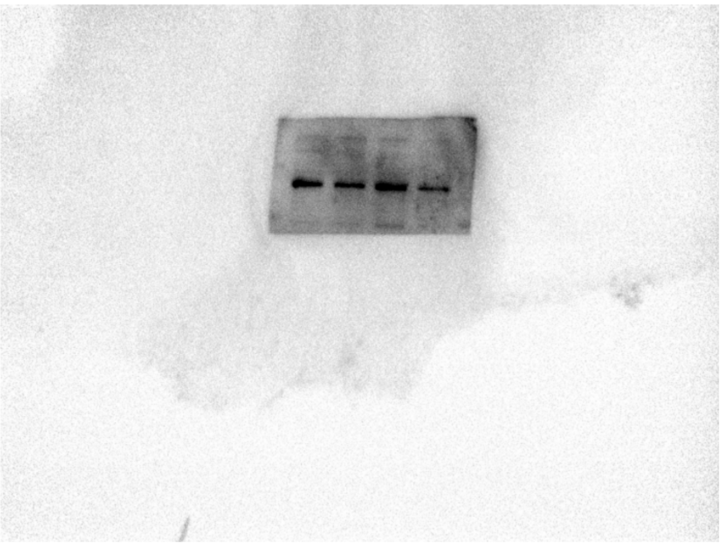

Figure 3D p62

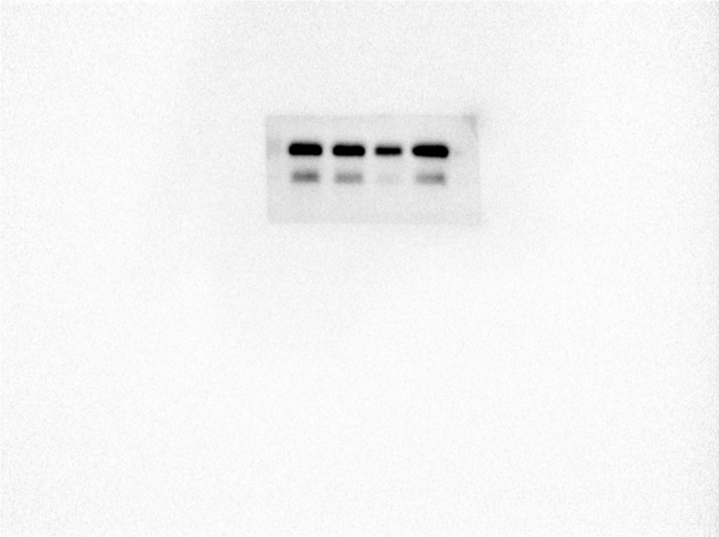

Figure 3D Beclin-1

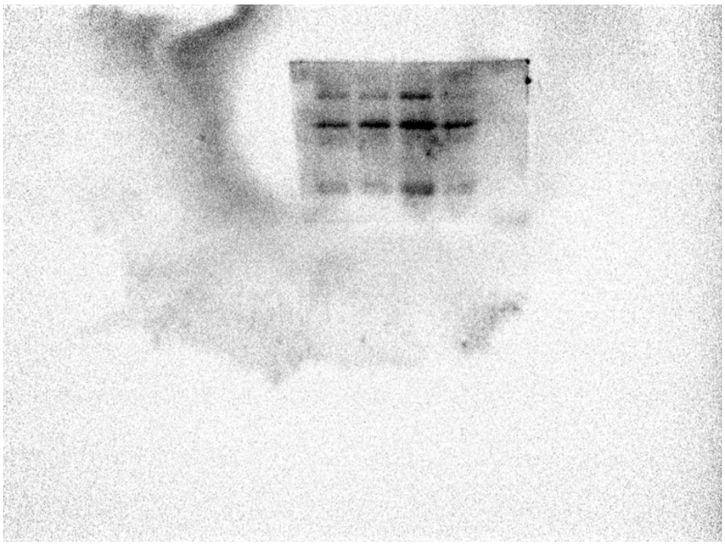

Figure 3D LC3B

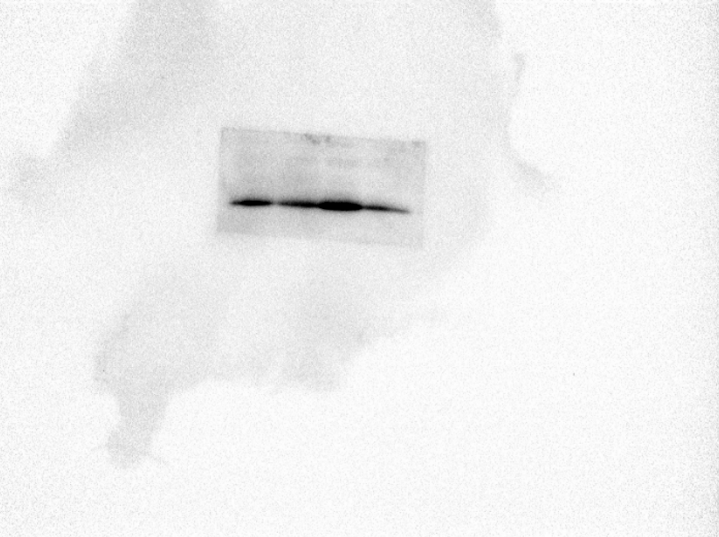

Figure 4C control--control--SDF-1α--SDF-1α

Figure 4C β-actin

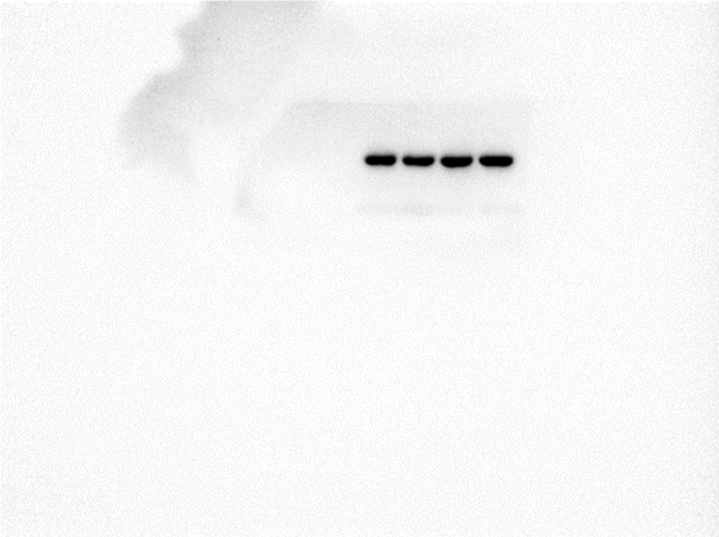

Figure 4C p-mTOR

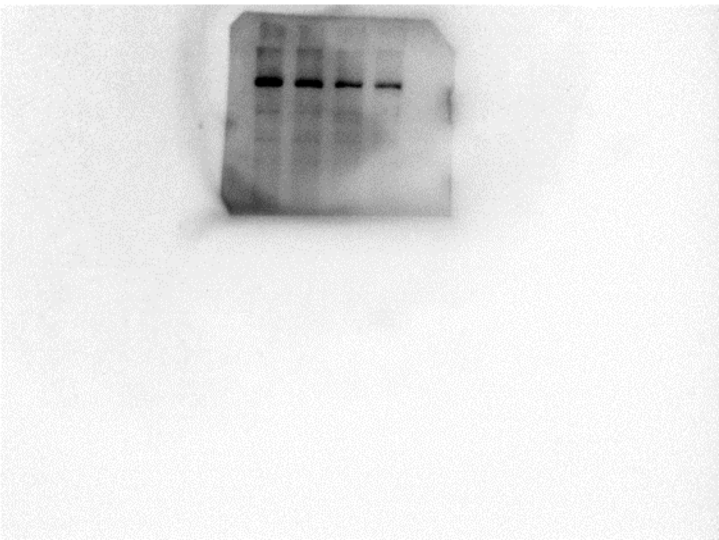

Figure 4C mTOR

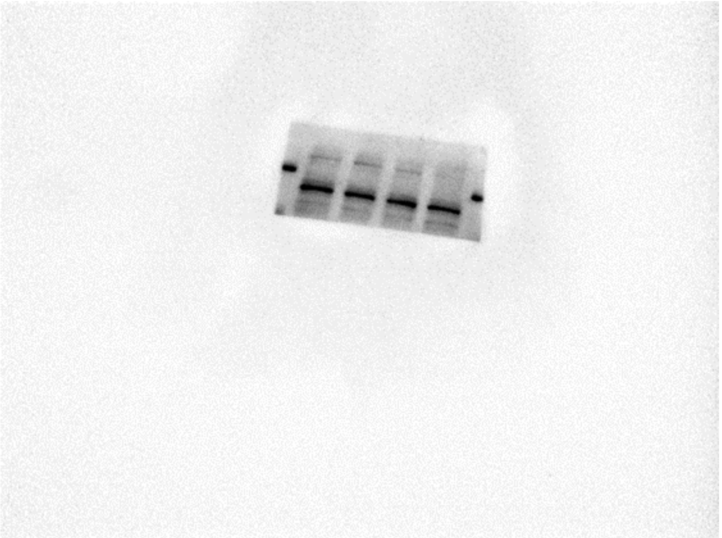

Figure 4E control--CQ--CQ+ SDF-1α(50--100--200 μg/ml)

Figure 4E β-actin

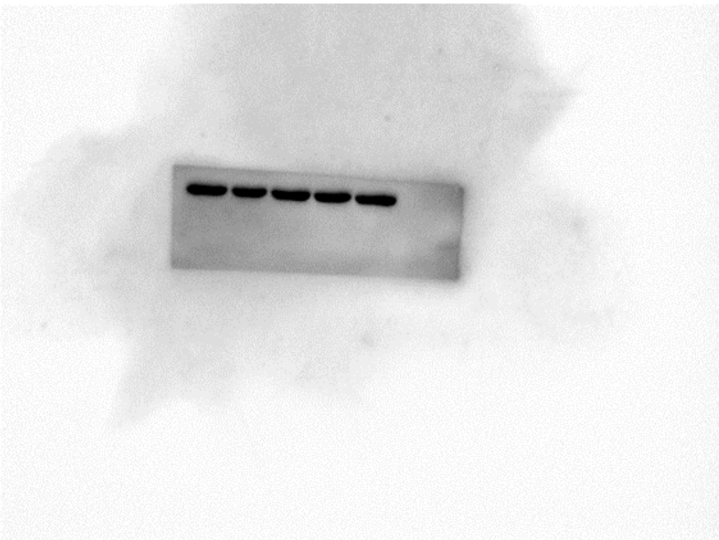

Figure 4E p-mTOR

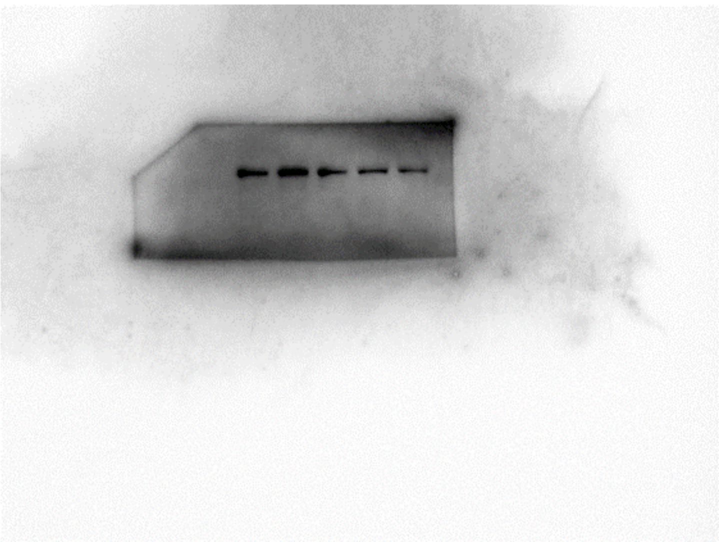

Figure 4E mTOR

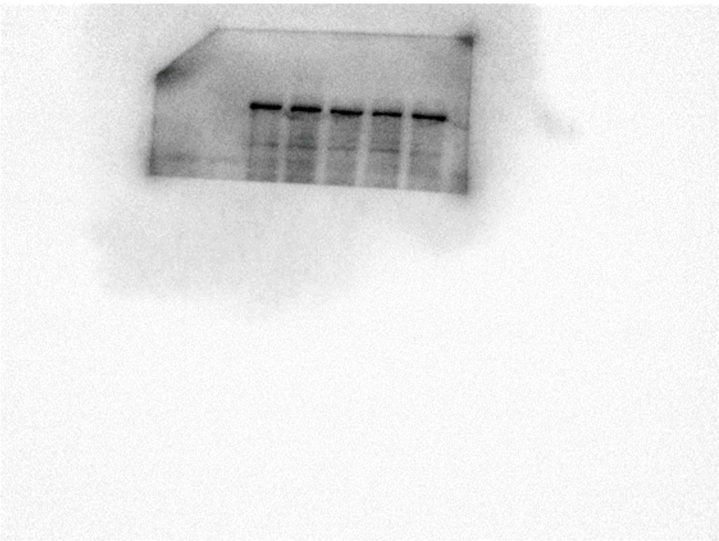

Figure 5A si-NC--si-CXCR4--si-NC+ SDF-1α--si-CXCR4+ SDF-1α

Figure 5A β-acin

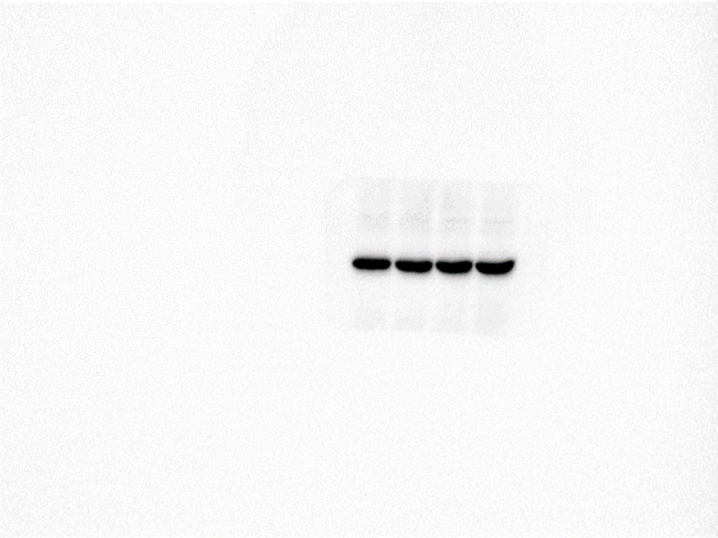

Figure 5A p-mTOR

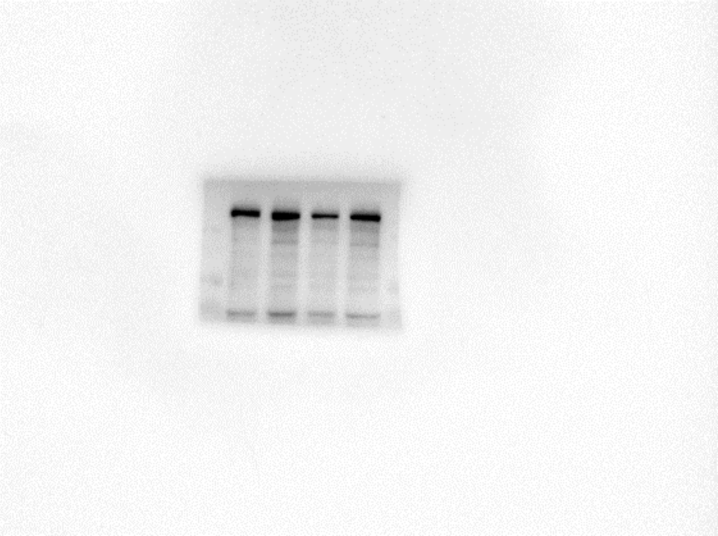

Figure 5A mTOR

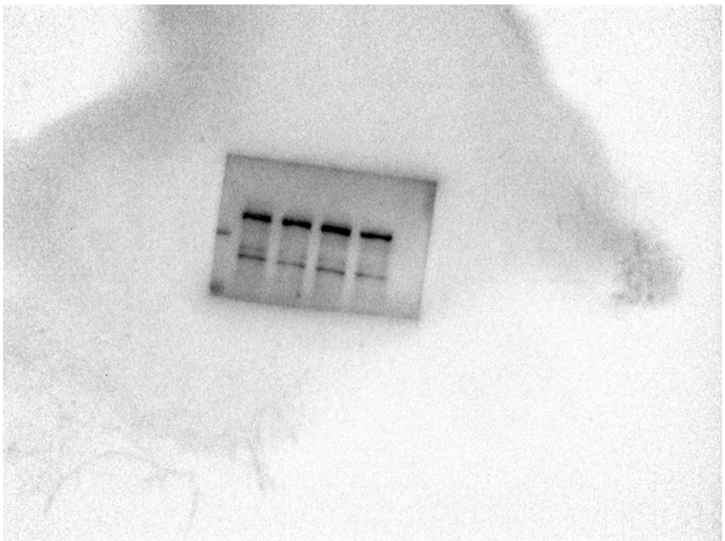

Figure 5E control--DMSO--SDF-1α--MHY1485+DMSO--SDF-1α+MHY1485+DMSO

Figure 5E β-actin

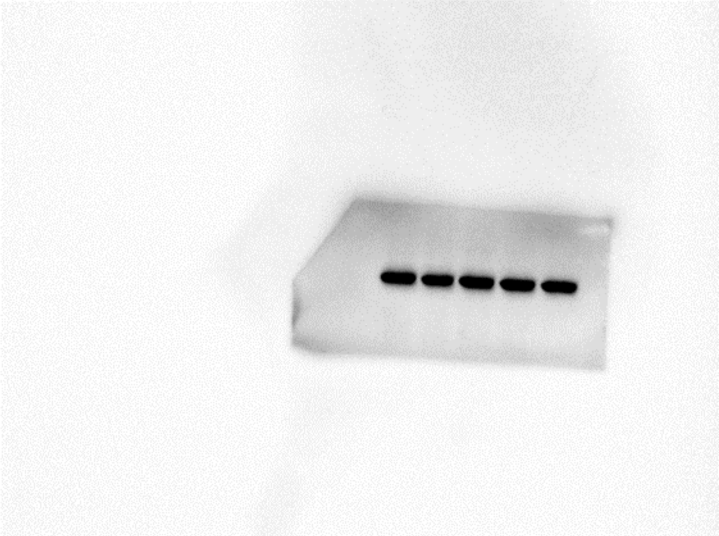
Figure 5E ULK-1

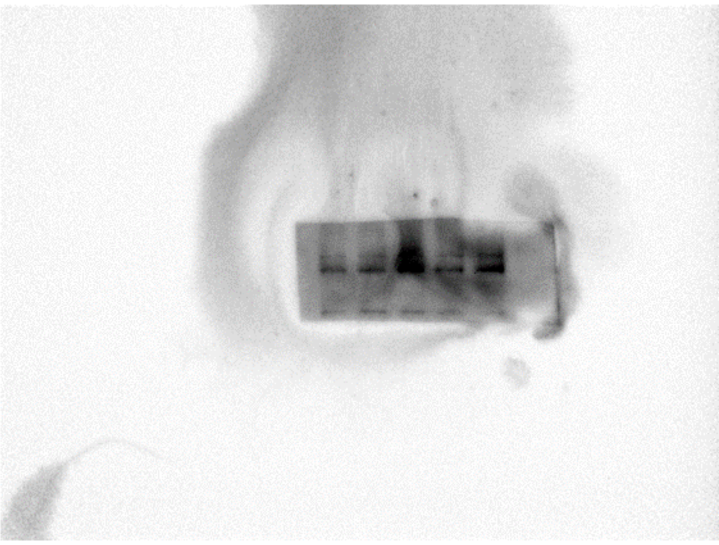
Figure 5E p62

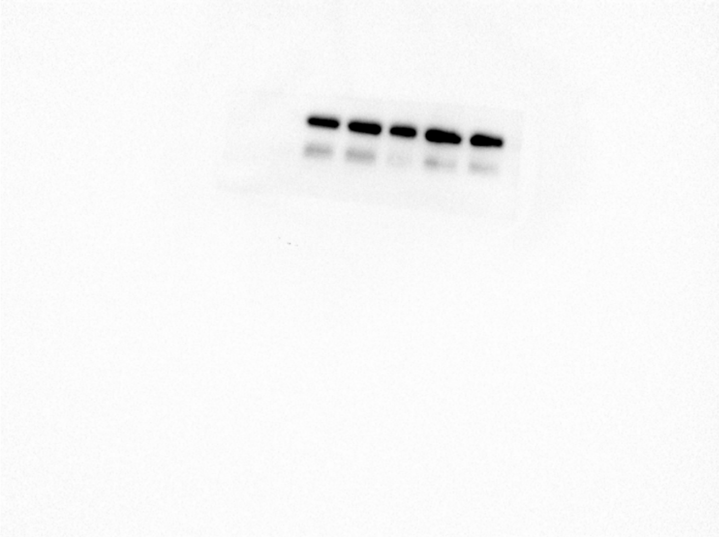
Figure 5E Beclin-1

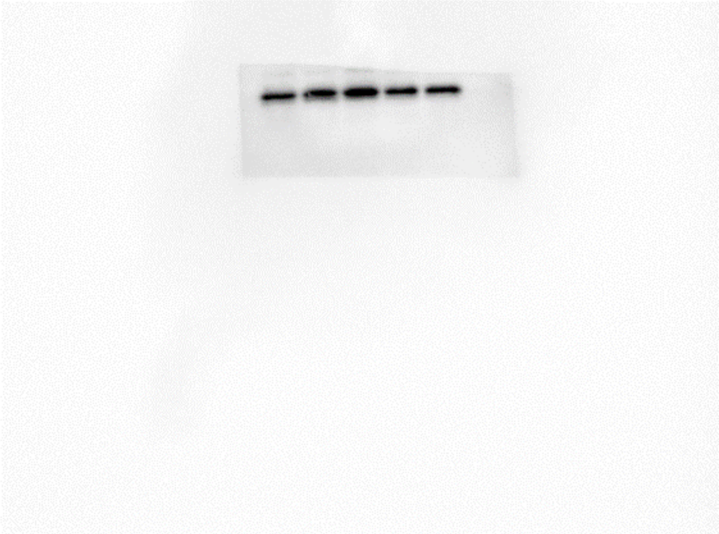

Figure 5E LC3B

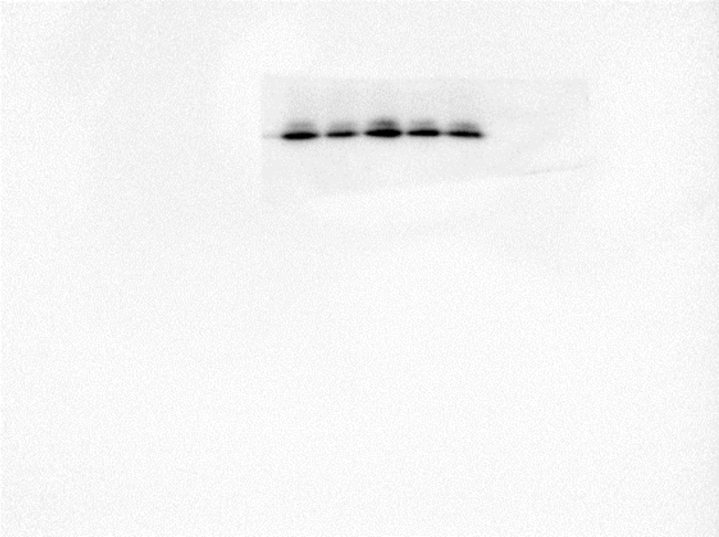

Figure S9

β-actin

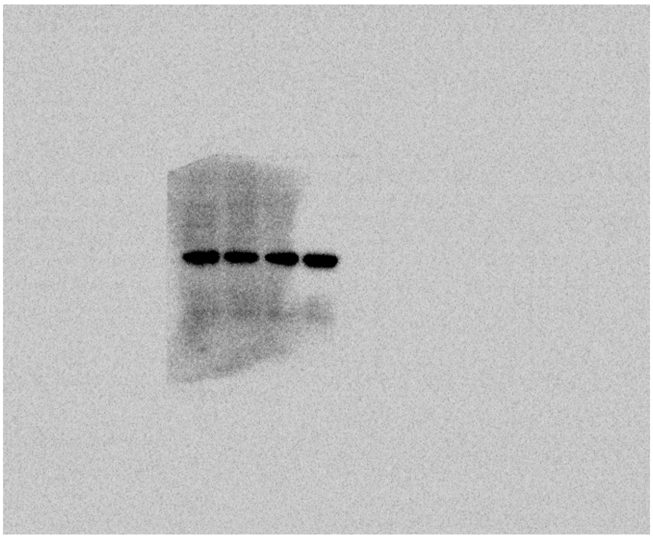

Col2a1

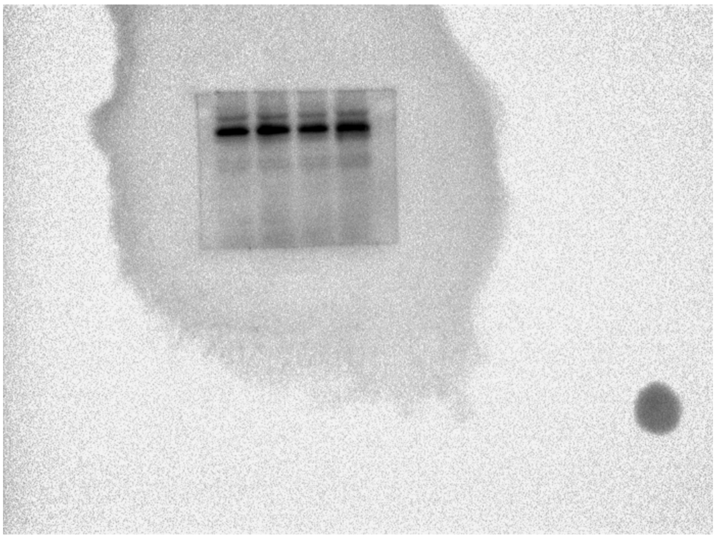

Aggrecan

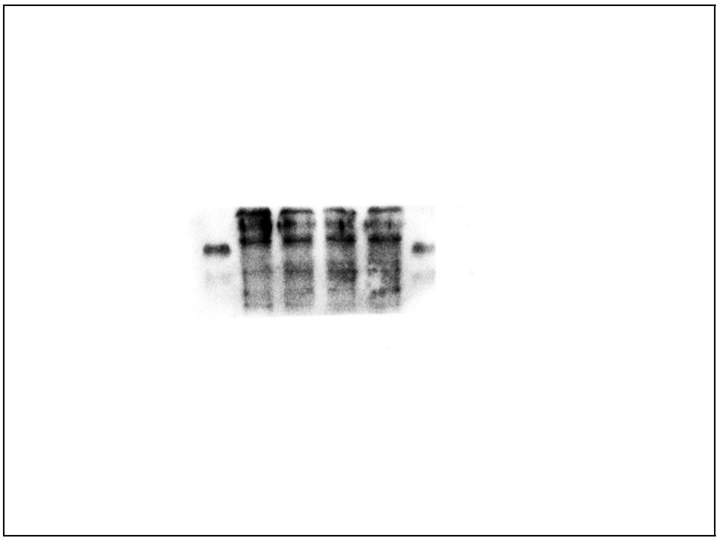

Supplement: Supplementary file 1 [file ijms-24-01710-s001.zip › ijms-2058409-supplementary.pdf]
